# High salt diet exacerbates colitis in mice by decreasing Lactobacillus levels and butyrate production

**DOI:** 10.1186/s40168-018-0433-4

**Published:** 2018-03-22

**Authors:** Pedro M. Miranda, Giada De Palma, Viktoria Serkis, Jun Lu, Marc P. Louis-Auguste, Justin L. McCarville, Elena F. Verdu, Stephen M. Collins, Premysl Bercik

**Affiliations:** 1 Farncombe Family Digestive Health Research Institute, Department of Medicine, McMaster University, Hamilton, Ontario Canada; 2 Graduate Program in Areas of Basic and Applied Biology, Instituto de Ciências Biomédicas Abel Salazar, Universidade do Porto, Porto, Portugal

**Keywords:** Salt, NaCl, Western diet, Colitis, Microbiota, Lactobacillus, Butyrate, MAPK-pathway

## Abstract

**Background:**

Changes in hygiene and dietary habits, including increased consumption of foods high in fat, simple sugars, and salt that are known to impact the composition and function of the intestinal microbiota, may explain the increase in prevalence of chronic inflammatory diseases. High salt consumption has been shown to worsen autoimmune encephalomyelitis and colitis in mouse models through p38/MAPK signaling pathway. However, the effect of high salt diet (HSD) on gut microbiota and on intestinal immune homeostasis, and their roles in determining vulnerability to intestinal inflammatory stimuli are unknown. Here, we investigate the role of gut microbiota alterations induced by HSD on the severity of murine experimental colitis.

**Results:**

Compared to control diet, HSD altered fecal microbiota composition and function, reducing *Lactobacillus* sp. relative abundance and butyrate production. Moreover, HSD affected the colonic, and to a lesser extent small intestine mucosal immunity by enhancing the expression of pro-inflammatory genes such as *Rac1*, *Map2k1*, *Map2k6*, *Atf2*, while suppressing many cytokine and chemokine genes, such as *Ccl3*, *Ccl4*, *Cxcl2*, *Cxcr4*, *Ccr7*. Conventionally raised mice fed with HSD developed more severe DSS- (dextran sodium sulfate) and DNBS- (dinitrobenzene sulfonic acid) induced colitis compared to mice on control diet, and this effect was absent in germ-free mice. Transfer experiments into germ-free mice indicated that the HSD-associated microbiota profile is critically dependent on continued exposure to dietary salt.

**Conclusions:**

Our results indicate that the exacerbation of colitis induced by HSD is associated with reduction in *Lactobacillus sp.* and protective short-chain fatty acid production, as well as changes in host immune status. We hypothesize that these changes alter gut immune homeostasis and lead to increased vulnerability to inflammatory insults.

**Electronic supplementary material:**

The online version of this article (10.1186/s40168-018-0433-4) contains supplementary material, which is available to authorized users.

## Background

The prevalence of chronic inflammatory diseases, such as multiple sclerosis (MS), rheumatoid arthritis, diabetes, and inflammatory bowel disease (IBD), has been steadily increasing over the past half-century [1]. Although multiple genetic factors have been identified to contribute to the risk of developing these disorders, genetic drift alone cannot explain the rapid increase in prevalence within this relatively short time interval [2]. Therefore, environmental factors, particularly related to dietary and hygiene habits, have been proposed as disease modifiers [3, 4]. The relationship between dietary habits and the increased prevalence of intestinal inflammatory disorders is of particular interest. Consumption of highly caloric processed foods, high in fat and simple carbohydrate content, and low in fiber content, has been steadily increasing in the “western world,” where the incidence of chronic inflammatory diseases is the highest [5, 6]. Strong epidemiological evidences support the concept that diet underpins the rise in chronic inflammatory diseases such as IBD, diabetes, or asthma [3, 5, 6]. However, the exact mechanisms by which specific dietary changes increase susceptibility to inflammatory diseases, and whether they are indeed mediated by changes in microbiota composition or function, are still unclear. Previous studies have shown that a high fat diet may have a direct pro-inflammatory effect, increasing the production of pro-inflammatory cytokines TNFα, IL-1β, and IL-6 [7]. Others have shown direct interactions between animal fat consumption, altered bile acid metabolism, and specific alteration in the gut microbiota, leading to increased colitis severity [8]*.* However, one characteristic element in the western diet that has been overlooked until recently is its high salt (sodium chloride, NaCl) content.

NaCl has been shown to induce pathogenic Th17 cells (IL-17-producing T-helper cells) in both human and mice naïve CD4^+^ cell cultures in vitro. Mice receiving a salt-enriched diet developed a more severe form of experimental autoimmune encephalomyelitis (EAE), an animal model of MS, mediated by IL-17A and RORγt expressing Th17 cells [9, 10]. Interestingly, high NaCl intake has been associated with increased disease activity in MS patients [11]. Moreover, a longitudinal clinical study showed that high NaCl intake increases circulating monocytes and levels of plasma cytokines such as IL-6 and IL-23 [12]. A recent study has shown that exposing human *lamina propria* mononuclear cells (LPMC) to high concentrations of NaCl enhances TNF-α and IL-17A release in a p38-dependent manner, and that feeding mice a salt-enriched diet exacerbates experimental colitis [13]. While all these studies suggest a promoting role of high salt diet in driving a pro-inflammatory state, the underlying mechanisms are still not fully understood.

We hypothesized that a high salt diet impacts inflammation via the interaction with the gut microbiota. In fact, it is well established that diet determines the composition [14–16] and function [17, 18] of the gut microbiota. It has been recently shown that diet low in dietary fiber may be causing irreversible changes in gut microbiota with some species disappearing over generations [19]. Given that gut microbiota composition has been implicated in the pathophysiology of multiple immune-related disorders, such as IBD, diabetes, or asthma [20], and taking into account that diet shapes the gut microbiota, we investigated the ability of high salt diet (HSD) to modulate gut microbial composition and metabolism, and to influence vulnerability to intestinal inflammatory stimuli.

## Methods

### Mice

Six- to eight-week-old specific pathogen-free (SPF) male C57BL/6 mice (obtained from Taconic) were housed at the animal facility of McMaster University under 12 h light/dark cycles and standard conditions for temperature and humidity. All mice received irradiated control diet (7004, Teklad, with 20% of calories from protein, 29% from fat, and 51% from carbohydrates, containing 0.4% Na and 0.7% Cl) and sterile tap water ad libitum upon arrival. Experiments started 1 week after arrival in the central animal facility to allow the animals to habituate to the novel environment. Mice were then divided into two groups: (1) control diet—mice were fed with control diet for the entire course of the experiment; (2) HSD (high salt diet)—mice received the same diet supplemented with 4% NaCl (custom ordered from Teklad), plus 1% NaCl sterile water, ad libitum, during 4 weeks. For germ-free (GF) mice experiments, C57BL/6 mice were re-derived in McMaster Axenic Gnotobiotic Unit (AGU) by two-stage embryo transfer and kept in dedicated isolators during the entire course of the experiment. For the microbiota-transfer experiment, GF mice were colonized by oral gavage with 200 μl of cecal microbiota from a mouse fed with control diet or HSD and housed in dedicated individually ventilated racks.

### DNA isolation and 16S rRNA Illumina sequencing

DNA was extracted from 100 mg of fecal content samples using the MagMAX DNA Multi-sample kit and the MagMAX Express-96 well magnetic particle processor (Thermofisher, Waltham, MA, USA) to achieve an automatic and standardized DNA extraction across samples. Briefly, fecal samples were mechanically homogenized with 2.8 mm ceramic beads (MoBio, Carlsbad, CA, USA; an additional 0.2 g of 0.1 mm glass beads were added), 100 μl of guanidine thiocyanate-EDTA-N-lauroyl sarcosine, and 800 μl of 200 mM NaPO_4_. Samples were homogenized for 3 min at 300 rpm and were centrifuged 5 min at maximum speed. 200 μl of the extract was added to the MagMAX Express plate for further DNA extraction procedures according to manufacturer’s instructions. Isolated DNA was kept at − 20 °C (or − 80 °C for longer storage). Polymerase chain reaction (PCR) amplification of the variable 3 (V3) region of the 16S rRNA gene was performed as previously described [21, 22]. Purified PCR products were sequenced using the Illumina MiSeq platform by the McMaster Genomics facility. Of note, we lost two samples during processing; one from baseline group and one from 4 weeks HSD group. The 16S rRNA samples metadata table is available in Additional file 1.

### Illumina sequencing processing and analysis

Custom, in-house Perl scripts were developed to process the sequences after Illumina sequencing [23]. The sl1p pipeline is open source and publicly available at https://bitbucket.org/fwhelan/sl1p. Briefly, Cutadapt [24] was used to trim any over-read, and paired-end sequences were aligned with PANDAseq [25], with a 0.7 quality threshold. If a mismatch in the assembly of a specific set of paired-end sequences was discovered, they were culled. In addition, any sequences with ambiguous base calls were also discarded. Operational taxonomic units (OTUs) were picked using AbundantOTU+ [26], with a clustering threshold of 97%. Taxonomy was assigned using the Ribosomal Database Project (RDP) classifier v.2.2 [27] against the Greengenes SSU reference database (2013 release) [28]. For all downstream analysis, we used Quantitative Insights Into Microbial Ecology (QIIME) [29]. We obtained a total of 2,839,134 reads after quality filtering, with an average of 76,733.351 reads/sample (range 34,470 to 144,746). OTU tables were filtered to exclude “root” and any OTU with less than 10 sequences in all samples, and finally rarefied to 30,000 reads/sample (OTU table is available in Additional file 1). Calculations of within-community diversity (α-diversity), between-community diversity (β-diversity), relative abundance taxonomic summaries, and the different statistical analyses were preformed using QIIME (scripts “alpha_rarefaction.py” and “beta_diversity_through_plots.py,” respectively). The relationships between HSD and microbiota phylogeny were explored using Principal Coordinate Analysis on unweighted UniFrac metrics and abundance Jaccard index (“beta_diversity_through_plots” script, QIIME), and the dissimilarity distance between the diet groups was tested using ANOSIM statistical method (“compare_categories” script, QIIME). For relative abundance taxonomic summaries and further statistical analysis, OTUs with a representation of less than 0.1% in the community were excluded. The correlations between fecal microbiota composition (at the highest taxonomic levels (family and genus) and at OTU level) and the diet were assessed by Spearman’s rank correlation coefficient with fisher *z*-transformation *p* value assignment method, using the “observation_metadata_correlation” script (QIIME), based on the attribution of a ranked value to the variable “Weeks of NaCl in diet”, from “0” to “4.” The “wash out” group was ranked “0.” PICRUSt [30] was used to predict the altered KEGG pathways based on the 16S sequencing data. L3 KEGG pathways correlation with length of HSD was calculated based on Spearman’s correlation using Prism GraphPad 6 (GraphPad software). Heatmaps were generated using the Heatmaps2 package (gplot) for R (version 3.3.2) (See R code script in Additional file 2). In the microbiota-transfer experiment, the OTUs were considered successfully transferred when present with an average of 2 reads or higher.

### Short-chain fatty acids (SCFA) quantification

SCFA were quantified as previously published [31, 32]. Briefly, 20–50 mg of frozen cecal content was collected into a tube; previously treated with methanol. The cecal samples were diluted 1:1 (*w*:*v*) with HCl 3.7% (10× diluted) and homogenized. Then 10 μl of internal standards and 500 μl of diethyl ether were added to the samples, and the tubes vortexed for 15 min. The extract (400 μl) was transferred to a clean tube. This step was repeated until we obtained a total of 800 μl of diethyl ether-fecal extract, 60 μl of which was then transferred to a chromatographic vial with 20 μl of N-tert-butyldimethylsilyl-N-methyltrifluoroacetamide (MTBSTFA). The organic extract-MTBSTFA was incubated at room temperature for at least 1 h, before being analyzed using the GC-MS Agilent 6890 N GC coupled to Agilent 5973 N Mass Selective Detector, with the column DB-17HT (30 m × 0.25 mm ID, 0.15 mm film).

### Experimental colitis

Additional groups of 6- to 8-week-old specific pathogen-free (SPF) male C57BL/6 mice (obtained from Taconic) and NIH Swiss mice (obtained from Harlan) were housed at the animal facility of McMaster University as described above. After a period of 4 weeks on HSD or control diet, mice were given 3.5% dextran sodium sulfate (DSS, MW 36-50 kDa; MP Biomedicals, Santa Ana, CA, USA) in drinking water for 5 days followed by regular water for the next 2 days. HSD mice were switched to control diet before starting DSS administration. Body weight and general health condition were assessed daily. At day 7, mice were euthanized and the colon was blindly evaluated using a previously validated score [33, 34]. Briefly, the colon was evaluated for muscle thickness, hyperemia and erythema, presence of blood in the stools, and stool formation (For details, see Additional file 2). Control mice (no DSS) received only water during the entire procedure. For germ-free (GF) mice, the DSS dose had to be lowered to a 2% solution due to their higher sensitivity to experimental colitis. For the microbiota adoptive transfer experiments in ex-germ free mice a 2.5% DSS solution was used, starting 5 days after colonization.

For Dinitrobenzene sulfonic acid (DNBS)-colitis, mice were anesthetized with isoflurane (3%) and were injected with 1 ml of saline subcutaneously. A custom-made polyester catheter was introduced 3.5 cm into the rectum and 100 μl of 50% EtOH containing 3.5 mg of DNBS (MP Biomedicals, Santa Ana, CA, USA) was injected. From that day, all mice received control diet with normal water containing 6% sucrose to prevent dehydration. At day 3 post-DNBS, mice were euthanized and blindly evaluated using a validated DNBS-colitis macroscopic score [35]. The colon was evaluated for adhesion to surrounding tissue, thickness, hyperemia, erythema, and stool consistency. Mucosal ulcers were quantified and measured (for details see Additional file 2). Mice from control group (no DNBS-colitis) were rectally injected with a 50% EtOH solution.

### Tissue processing

Blood was collected via orbital bleeding and centrifuged at 5000 rpm for 10 min. The serum was separated and stored at − 80 °C. The colon (full length, from the rectum to the cecum) was excised and measured. Colonic content was removed and 0.5–1.0 cm sections were dissected from mid-colon and either snap-frozen in liquid N_2_ for MPO analysis, stored in RNA later (Ambion) for RNA extraction, or fixed in 10% buffered formalin for histology assessment (from the border of an ulcer when present). MPO was performed on frozen tissues as previously described [36], and its activity expressed in units per milligrams of tissue. For histology, colonic sections were embedded in paraffin, 48 h after being fixed in 10% formalin solution, and were stained with hematoxylin and eosin (H&E). Inflammation was blindly evaluated following an inflammation histology scoring system (see Additional file 2).

### Gene expression

Total RNA was extracted and purified using RNeasy Mini Kit (Qiagen, Hilden, Germany) according to manufacturer’s instructions, including the DNA removal step using the RNase-Free DNase Set (Qiagen). NanoString nCounter Gene Expression CodeSet for mouse inflammation genes (v2; 248 genes) and a custom CodeSet panel that included 68 genes related to immune, gut barrier, and neurobiology functions (see Additional file 2) were run according to manufacturer’s instructions (NanoString Technologies Inc., Seattle, WA, USA). Data from nCounter was analyzed using nSolver 2.5 software (Nanostring Technologies Inc.) (Nanostring raw counts are provided in Additional file 3). The log2 ratios of gene expression were then uploaded into Ingenuity Pathway Analysis software (Qiagen) for further analysis. For Q-PCR gene expression, cDNA library was generated using the SuperScript III First-Strand Synthesis System (Invitrogen, Carlsbad, CA, USA), according to manufacturer’s instructions, using Oligo-dT primers (Qiagen). Additional information on the primers is provided in Additional file 2.

### Immune cells extraction

Mesenteric lymph nodes (mLN) were harvested aseptically, and the cells were released from the nodes using a 70-μm strainer with the help of a syringe plunger, and were suspended in RPMI medium. After washing, the cells were kept in complete RPMI medium on ice until further use.

Colonic and small intestinal (SI) *lamina propria* (LP) cell extraction was performed using established protocols [37]. Briefly, colon and SI were removed and kept in cold sterile Dulbecco’s phosphate-buffered (DPBS) solution until further processing. All fatty tissue, content, and the payer’s patches were removed. Sections of intestine were open longitudinally, were cut into 0.5–1 cm pieces, and were incubated in 2 mM Dithiothreitol (DTT)/Hank’s solution (HBSS) on a horizontal shaker (240 rpm) for 15 min at 37 °C to remove the mucous layer. After vigorous vortexing, the tissues were strained with a sterile metal strainer and were incubated with a 5 mM EDTA/HBSS solution for 10 min at 37 °C (240 rpm) to remove the epithelial layer. After vigorous vortexing, the tissues were strained once again. This epithelial removal step was repeated 3–4 times. After removing the EDTA solution, the tissues were digested in 0.1% Collagenase solution (type VIII; Sigma-Aldrich, St. Louis, MO, USA) and DNAse (I recombinant, grade I; Roche, Basel, Switzerland; 0.05 mg/50 ml of digestion solution) to aid the release of the LP cells (15 min for SI and 25 min for colon, 37 °C). Supernatants were strained through a 40-μm cell strainer to separate LP cells from the remaining intestinal tissue and were washed thoroughly. The cell suspension was then purified by Percoll (GE Healthcare, Chicago, IL, USA) density gradient method. Briefly, the cell suspension was re-suspended in 35% Percoll solution and underlayed with a solution of 70% Percoll. The tubes were centrifuged at 4 °C, 670 G, for 30 min (without break and with slow acceleration). The purified cell suspension was removed from the interphase, was washed, and was resuspended in complete RPMI. For LP cells gene expression, the cell suspension was resuspended in RLT buffer (RNeasy Mini Kit, Qiagen) and was snap-frozen in liquid nitrogen.

### Flow cytometry

Cells isolated from mLNs, colon LP and SI LP were divided into two groups for intracellular staining: (1) anti-IL17A + anti-RORγt; and (2) anti-FoxP3. Cells of the first group were stimulated for 4–5 h at 37 °C with PMA (50 ng/ml), ionomycin (750 ng/ml; both from Sigma-Aldrich), in the presence of 10 μg/ml of Brefeldin A (BD Biosciences, San Jose, CA, USA), to activate IL17A expression. After this period, all cells were washed with PBS solution and were stained for cell viability with Fixable Viability Dye eFluor® 780 (1:1000; eBiosciences, San Diego, CA, USA) for 30 min at RT. Cells were then stained with the following extracellular antibodies: anti-TCRb-PerCP-Cy5.5 (1:100; H57-597 clone; eBioscience) and anti-CD4 + -Pacific Blue (1:200, RM4-5 close, BD Biosciences) for 30 min at 4 °C. Cells were fixed and permeabilized with FoxP3 Fixation/Permeabilization Concentrate and Diluent (eBioscience) according to manufacturer’s instructions and were stained for 90 min at 4 °C, with the following intracellular antibodies: anti-IL17A-APC (1:100; TC11-18H10.1 clone, Biolegend, San Diego, CA, USA) and anti-RORγt-PE (1:100; AFKJS-9 clone; eBioscience); or anti-FoxP3-FITC (1:100; FJK-16s clone; eBioscience). The data was acquired on LSR II cytometer (BD Bioscience) and was analyzed with FlowJo software (10.2 version) (TreeStar, Ashland, OR, USA).

### Statistical analysis

Data analysis was carried out using GraphPad Prism 6 (GraphPad Software, La Jolla, CA, USA). Student’s *t* test, multiple *t* test, one-way ANOVA, or two-way ANOVA were used as appropriate. For ANOVA, samples were corrected for multiple comparisons using Tukey’s test. For Nanostring data, including heatmaps, the statistical analysis was carried out using nSolver 2.5 software (Nanostring Technologies). Heatmaps were generated using Euclidean distance metric based on group average. *P* < 0.05 was considered as statistically significant. Values are presented as means ± SEM. The raw data of all experiments is available in Additional file 1. 

## Results

### HSD alters gut microbiota composition

**Fig. 1:**
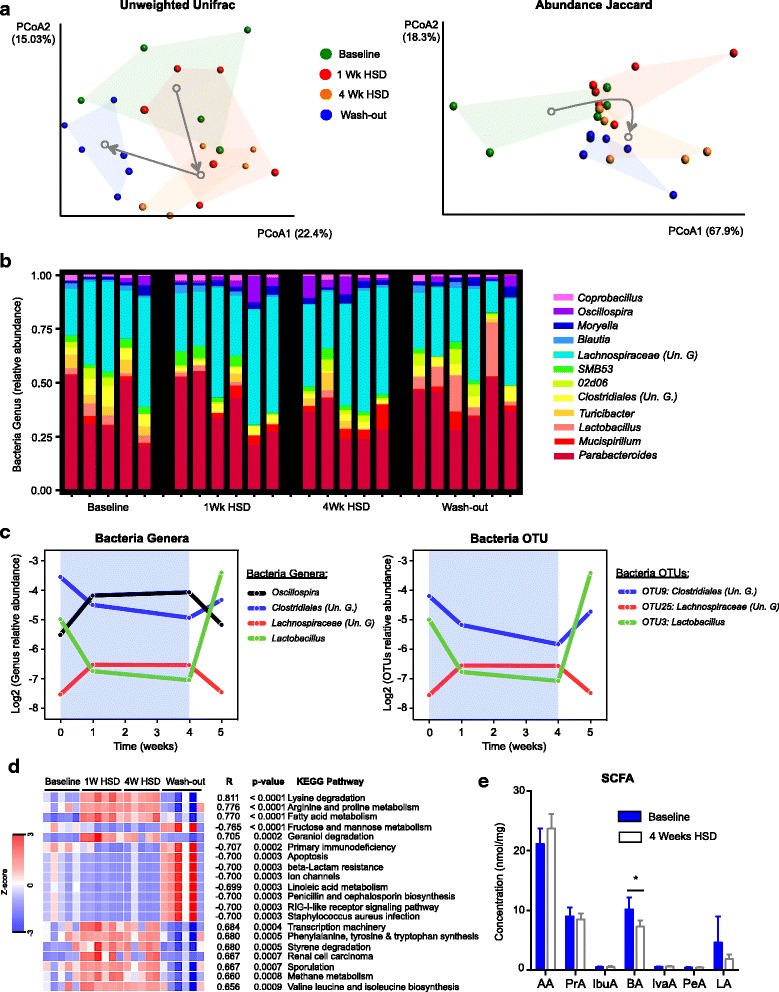
High salt diet alters gut microbiota composition and function. **a** Principal coordinate analysis (PCoA) plots of unweighted UniFrac metric (left panel) and abundance Jaccard index (right panel) of fecal microbiota composition over time exposed to HSD. Gray arrows indicate the direction of the significant microbiota composition that shifts between groups. **b** Summary of the relative abundance of microbial genera present in 99.9% of the community overtime exposure to HSD. **c** Relative abundance of fecal bacterial genera (left panel) and OTUs (right panel) that significantly correlate with HSD (*q* < 0.05). The blue background represents the period exposed to HSD. **d** Heatmap of the 20 most significant KEGG pathways that correlate with HSD in fecal microbiota. **e** SCFA quantification at 0 and 4 weeks after HSD. Un. G (Unclassified Genus). AA (acetic acid), PrA (propionic acid), IbuA (isobutyric acid), BA (butyric acid), IvaA (isovaleric acid), PeA (pentanoic acid), LA (lactic acid). Values are presented as means ± SEM. *n* = 5–6 mice/group. *p < 0.05

To investigate the impact of HSD on gut microbiota composition, we analyzed intestinal microbiota dynamics of mice fed with HSD for 4 weeks, with a follow-up at 1 week after the mice returned to the control diet. While fecal bacterial richness (observed species) and diversity (Chao1 diversity index) were not affected during the HSD, both α-diversity metrics decreased at 1 week follow-up (Additional file 4: Figure S1). Despite some variability within groups, we observed a significant modulation of fecal bacteria composition with time exposure to HSD, based on the phylogenetic distance metric unweighted UniFrac (UnwUF) and the non-phylogenetic abundance weighted Jaccard distance (AJac) (ANOSIM of UnwUF: *R* = 0.416, *p* = 0.001; ANOSIM of AJac: *R* = 0.283, *p* = 0.003) (Fig. 1a, Table 1). 1 week of HSD was not sufficient to significantly modulate the microbiota, but 4 weeks of HSD led to a significant shift in the composition, as revealed by ANOSIM pairwise analysis (UnwUF: *R* = 0.480, *p* = 0.004; AJac: *R* = 0.036, *p* = 0.008; Table 1). Interestingly, 1 week after returning to the control diet, fecal microbiota composition partially reverted to its original composition based on unweighted and weighted UniFrac metrics (Fig. 1a, Table 1 and Additional file 4: Figure S2).

**Table 1:** ANOSIM statistical analysis of distance metrics on the effect of HSD on fecal microbiota composition

	Unweighted UniFrac		Abundance Jaccard index	
	ANOSIM *R*	ANOSIM *p*	ANOSIM *R*	ANOSIM *p*
All groups	0.416	0.001	0.283	0.003
Baseline vs 1 week HSD	0.144	0.133	0.211	0.108
Baseline vs 4 weeks HSD	0.480	0.004	0.360	0.008
Baseline vs wash out	0.341	0.034	0.197	0.106
1 week HSD vs 4 weeks HSD	0.277	0.052	0.528	0.006
4 weeks HSD vs wash out	0.525	0.007	0.075	0.237

In order to better understand the impact of HSD on the microbial community, we studied the correlation between HSD exposure and the taxonomic composition at genus and OTU level. Four bacterial genera were found to significantly correlate with HSD (Fig. 1b, c, Additional file 4: Table S1). *Lactobacillus* genus showed the strongest correlation by decreasing 2-fold of its relative abundance after 4 weeks of HSD and quickly normalizing after returning to the control diet (*r* = − 0.703, *q* = 0.003). An unclassified genus belonging to the order *Clostridiales* also decreased relative abundance over 1-fold after 4 weeks of HSD and partially normalized 1 week after returning to control diet (*r* = − 0.605, *q* = 0.017). Finally, unclassified genera belonging to the *Lachnospiraceae* family and the *Oscillospira* genus showed a positive correlation with HSD (*r* = 0.614, *q* = 0.017 and *r* = 0.548, *q* = 0.042, respectively) (Fig. 1b, c, Additional file 4: Table S1). Similarly, three fecal OTUs correlated with time of exposure to HSD (Fig. 1c, Additional file 4: Table S2): OTU #3, belonging to the genus *Lactobacillus* (*r* = − 0.707, *q* = 0.005), OTU #9, belonging to the order *Clostridiales* (*r* = − 0.684, *q* = 0.005), and OTU #25, belonging to the family Lachnospiraceae (*r* = 0.609, *q* = 0.027).

To test the stability of fecal microbiota and rule out spontaneous changes in microbial profiles during this age period, we analyzed microbiota composition in an additional group of control mice fed with control diet. There were no spontaneous changes in the microbiota genus affected by HSD such as *Lactobacillus*, *Oscillospira* and unclassified genus from *Clostridiales* and *Lachnospisaceae*, between weeks 8 and 12, in mice on control diet (Additional file 4: Figure S3). However, the microbiota profiles of mice on HSD and control diet clustered separately at both time points, likely consequence of the cage effect and low number of mice (Additional file 4: Figure S3).

### HSD affects predicted microbial metabolic activity and luminal SCFA levels

To investigate whether the differences in fecal microbiota composition induced by HSD could impact its metabolic activity, we assessed inferred metagenomic KEGG pathways using PICRUSt [30]. Multiple pathways appeared to be affected, with three of them being strongly upregulated by HSD, including “fatty acid metabolism,” “lysine degradation,” and “arginine and proline metabolism,” while “fructose and mannose metabolism” was downregulated (Fig. 1d). As HSD altered the relative abundance of several OTUs and genera of major SCFAs producers such as *Lachnospiraceae*, *Clostridiales*, and *Oscillospira*, and PICRUSt predicted changes in the metabolism of fatty acids, we sought to quantify luminal SCFA in mice on HSD. Out of the seven SCFA tested, only butyrate levels were significantly affected, showing a decrease after 4 weeks of HSD (Fig. 1e). We also observed a decrease in lactic acid concentration; however, this change did not reach statistical significance. Taken together, these results show that HSD modulates both composition and function of the gut microbiota.

### HSD modulates colonic and small intestinal immune gene expression

**Fig. 2:**
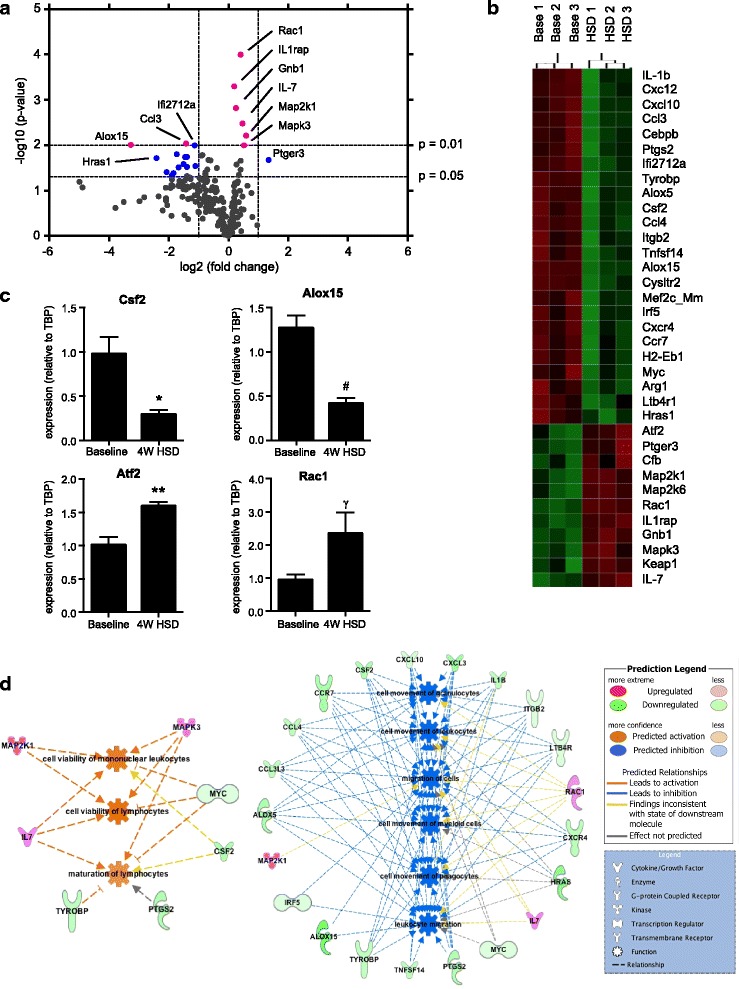
High salt diet modulates immune expression in colonic *lamina propria*. **a** Volcano plot of gene expression in colonic *lamina propria* total cell extract of mice receiving HSD or control diet for 4 weeks, *n* = 9 mice/group. Pink dots: genes with *p* < 0.01. Blue dots: genes with 0.01 < *p* < 0.05, fold change > 2. **b** Heatmap of immune genes that changed with HSD (*p* < 0.05). Based on group average, Euclidean distance metric. **c** qRT-PCR analysis of differentially expressed genes in the two groups. Values are presented as means ± SEM, *n* = 5–6 colon/group, *p < 0.05, **p < 0.01, #*p* < 0.001, γ*p* = 0.065. **d** Gene-cell function networks obtained from Ingenuity Pathway Analysis. On the left, network with the genes that contribute to the predicted inhibition of functions related to immune cell migration, and, on the right, network with the genes that contribute to the predicted upregulation of the immune cell viability and maturation

We subsequently tested whether the HSD-induced changes in gut microbiota were affecting host’s gut mucosal homeostasis. We assessed gene expression profile of colonic *lamina propria* immune cells with nCounter Mouse Inflammation V2 Panel (Nanostring). HSD altered the expression of 35 genes (*p* < 0.05) in mouse colonic *lamina propria* cell extract, with 11 transcripts being significantly upregulated and 24 being downregulated (Fig. 2a, b; See Additional file 4: Table S3 for the full list of affected genes). Some of these genes were selected for validation by q-PCR (Fig. 2c). A considerable part of the affected transcripts were chemokines, cytokines, or their receptors (Additional file 4: Table S3). Among these, the cytokine IL-7 and the receptor IL-1rap were upregulated by HSD (0.47 and 0.19 log2 ratio, respectively), while all other cytokines and chemokines were downregulated. Of note, we observed the downregulation of several chemokines (and chemokine receptors) important for the chemotaxis of monocytes/macrophages, dendritic cells, neutrophils, and NK cells, such as Ccl3, Ccl4, Cxcl10, Cxcl2, Ccr7, and Cxcr4 (Additional file 4: Table S3), indicating a possible impairment of their migration. The most significant change observed was the upregulation of Rac1 (Ras-related C3 botulinum toxin substrate 1) transcript (log2 ratio = 0.41, *p* = 0.0001), a member of Rho family GTPases with broad functions, from innate immunity processes to leukocyte chemotaxis and barrier function.

In order to identify possible functional pathways affected by HSD we integrated our gene expression data with publicly available databases results using Ingenuity Pathway Analysis (IPA) bioinformatics platform. The upregulation of the genes Map2k6, Map2k1, Mapk3, Atf2, and IL1rap was predicted by IPA to stimulate several pro-inflammatory canonical pathways such as “acute phase response signaling,” IL-6, and Mapk signaling (Additional file 4: Table S4). The roles of macrophages and IL-17 signaling were also strongly predicted to be affected by HSD (Additional file 4: Table S4 ). Even though IL-17A cytokine itself was not found to be significantly affected by HSD in *lamina propria* colonic cell extracts, IPA identified multiple genes related to IL-17 signaling to be affected, such as Mapk3, Map2k1, Map2k6, and the transcription factor Atf2 (all upregulated), as well as Cxcl10, Hras, and Ptgs2 (all downregulated). When analyzing functions affected by HSD in the colon, IPA predicted downregulation of leukocyte migration and movement (Fig. 2d, Additional file 4: Table S5). This was expected since HSD suppressed the expression of many chemokines and cytokines responsible for immune cells chemotaxis. On the other hand, the functions related to leukocyte viability and maturation were predicted to be upregulated, based on the expression of genes like IL-7, Map2k1, and Mapk3 (Fig. 2d, Additional file 4: Table S5).

Although the focus of our study was the effect of HSD on colonic microbiota and immune function, we also assessed its effect on small intestine recognizing that that most of the Na^+^ and Cl^−^ is absorbed in this compartment. We observed a significant change in the expression of 11 genes (*p* < 0.05), including the upregulation of IL17A and Defa-rs1, as well the downregulation of IL12a and Cxcr4 (Additional file 4: Figure S4 and Table S6). Only two genes (Cxcr4 and Mef2c_Mm) were affected similarly in both colonic and small intestinal *lamina propria* cell extracts, emphasizing the distinct nature of the immune milieu of each gut compartment. IPA identified three canonical pathways related to bacteria recognition, toll-like receptors and IL-17 signaling to be most affected in the small intestine (Additional file 4: Table S7). While “recruitment of leukocytes” (*z* = 0.196, *p* = 4.01E-05) was upregulated, “proliferation of lymphocytes” (*z* = − 2.398, *p* = 5.44E-06) was downregulated.

### HSD increases RORγt^+^IL17^+^ T-cells in mLNs but not in intestinal lamina propria

To better understand the effect of HSD on lymphocyte function, we phenotyped T-cell populations in the small intestinal and colonic *lamina propria* and in mesenteric lymph nodes (mLNs) using flow cytometry. HSD led to a 2-fold increase of RORγt^+^IL17A^+^ cells in the mLNs but not in small intestinal or colonic *lamina propria* (Additional file 4: Figure S5A). However, we consistently observed a mild but not statistically significant increase of this cell population in the colon across all experiments. No change was observed in the expression of IFNγ by CD4+ T-cells in mice on HSD (data not shown), nor in the frequency of CD4^+^FoxP3^+^ T-cells, even though, for the latter, we noticed a small increase of this cell type across tissues in all experiments (Additional file 4: Figure S5B). Taken together, these results show that HSD affects the gut mucosal immune compartment.

### HSD exacerbates DSS colitis

**Fig. 3:**
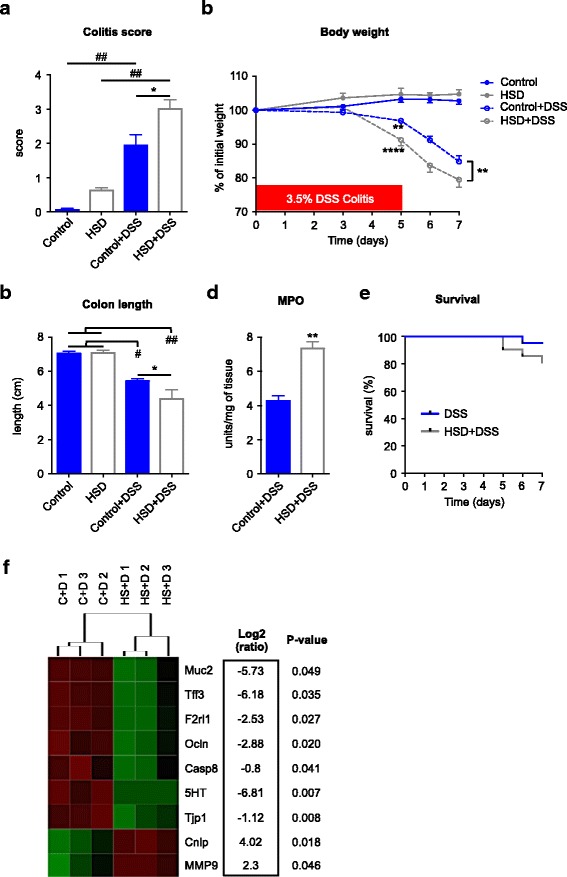
High salt diet exacerbates DSS colitis. **a** DSS colitis macroscopic scores at endpoint (day 7) in four groups of mice: control (control diet, no DSS), HSD (high salt diet, no DSS), control+DSS (control diet, DSS in water), HSD + DSS (high salt diet, DSS in water). **b** Body weight changes during DSS administration. **c** Colon length at endpoint. **d** Colonic MPO of the two groups of mice receiving DSS. **e** Survival curve of the two groups of mice with DSS colitis. **f** Heatmap of differentially expressed genes (*p* < 0.05) in the colon of mice with DSS colitis: C + D (control diet+DSS), HS + D (HSD + DSS). The heatmap was generated based on group average using Euclidean distance metric. Colitis scores, weight change, colon length, and MPO are presented as means ± SEM; *n* = 8–9 mice/group. *p < 0.05, **p < 0.01, #*p* < 0.001, ##*p* < 0.0001

To investigate how the changes in gut microbiota and immune function affect vulnerability to inflammatory insults, we used DSS and DNBS colitis models. We confirmed that mice on HSD developed a more severe DSS colitis when compared to mice on control diet, as previously described [13], characterized by an increased rectal bleeding, diarrhea, and general inflammation of the colonic tissue (Fig. 3a). HSD induced higher weight loss on day 5 of DSS administration, when compared to DSS mice on control diet, a difference that was maintained until the end of the experiment, on day 7 (Fig. 3b), indicating an accelerated onset and increased severity of colitis. Colon shortening, normally used as indicator of colitis severity, was greater in HSD mice compared to control diet mice (Fig. 3c). MPO activity, reflecting neutrophil infiltration, was increased 2-fold in the colon of mice on HSD after DSS administration when compared to mice on control diet (Fig. 3d). Furthermore, there was a significant difference in mortality between the two groups, with 5% of mice on control diet meeting the endpoint criteria prematurely, while this number increased to 19% in mice on HSD (Fig. 3e).

To better understand the immune responses during DSS colitis, we assessed the expression of a group of genes related to immune and barrier function using a customized Nanostring gene expression codeset (see list of genes in Additional file 2). HSD altered the expression of nine genes in the colon of mice on DSS, when compared to DSS mice on control diet (Fig. 3f). HSD decreased expression of genes involved in the maintenance of mucosal barrier such as Tjp1 (tight junction protein ZO-1), Muc2 (Mucin 2), Tff3 (trefoil factor 3), and Ocln (Occludin). On other hand, HSD mice had an increased expression of MMP9 (Matrix Metalloproteinase 9) and Cnlp (Cathelcidin-related antimicrobial peptide), genes expressed by innate immune cells such neutrophils and macrophages and previously described to be over-expressed in gut inflammatory conditions [38, 39]. HSD also reduced expression of two genes strongly expressed by epithelial cells: F2rl1 (protease activated receptor 2) and 5-HT (serotonin). This finding might be a consequence of the severe epithelial damage observed in colitis. Thus, the altered gene expression profile supports the notion that HSD exacerbates DSS colitis.

These results were reproduced using another mouse strain; NIH Swiss mice also developed a more severe DSS colitis after 4 weeks of HSD, confirming that HSD induces susceptibility to experimental colitis in multiple mouse strains (Additional file 4: Figure S6).

### HSD exacerbates DNBS-colitis

**Fig. 4:**
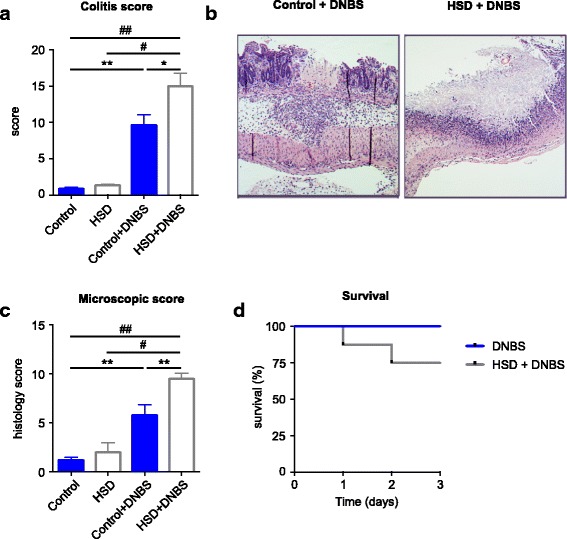
High salt diet exacerbates DNBS colitis. **a** DNBS colitis macroscopic scores at endpoint (day 3) in four groups of mice: control (control diet, 50% EtOH vehicle), HSD (high salt diet, 50% EtOH vehicle), DNBS (control diet, DNBS in 50% EtOH vehicle), HSD + DNBS (high salt diet, DNBS in 50% EtOH vehicle). **b** Representative H&E-staining of colonic section of DNBS mice highlighting the ulcers in the mucosa. **c** Histological scores in colonic sections. **d** Survival curve of the two groups with DNBS colitis. Colitis and histology scores are presented as means ± SEM; *n* = 5–9 mice/group. *p < 0.05, **p < 0.01, #*p* < 0.001, ##*p* < 0.0001

To confirm the increased sensitivity to colonic inflammation in mice on HSD, we used the DNBS model. Similar to the DSS colitis model, we chose a concentration of DNBS that would induce a mild form of colitis (3.5 mg of DNBS/mouse), in order to appreciate the effect of diet on worsening of experimental colitis. Similar to the results obtained with DSS-induced colitis, mice that were previously exposed to a 4-week HSD developed more severe colitis after rectal injection of DNBS (Fig. 4a). HSD mice had higher number of ulcers that were larger in size, when compared to control diet mice. Figure 4b, c shows that HSD mice developed ulcers that affected the entire colonic wall, up until the serosa with large clusters of cellular infiltrates, and, in some cases, a total loss of the epithelial layer. Strikingly, while none of the control diet mice met the endpoint criteria to be euthanized at this mild DNBS concentration, the mortality of HSD mice after DNBS administration reached 25% (Fig. 4d). Taken together, these results show that HSD exacerbates both DSS- and DNBS-colitis.

### HSD exacerbates experimental colitis in a gut microbiota-dependent manner

**Fig. 5:**
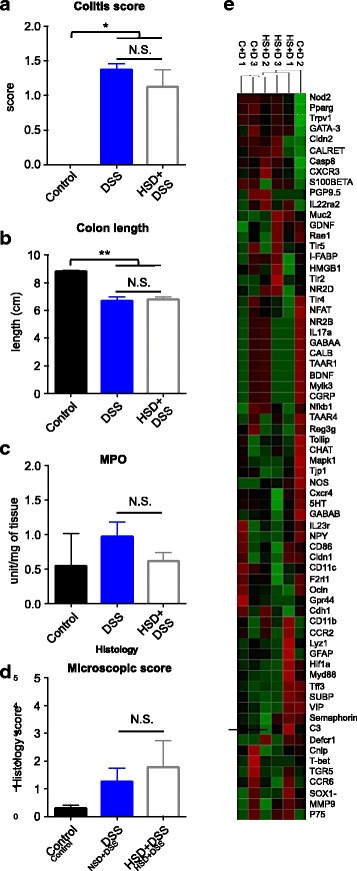
The effect of high salt diet on exacerbation of colitis is dependent on gut microbiota. **a** DSS colitis macroscopic scores at endpoint (day 7) in three groups of mice: control (no DSS colitis), DSS (control diet, DSS in water), HSD + DSS (high salt diet, DSS in water). **b** Colon length of mice at endpoint. **c** Colonic MPO. **d** Histological scores of the DSS colitis. Values are presented as means ± SEM; *n* = 3–6 mice/group. *p < 0.05, **p < 0.01. **e** Heatmap of the gene expression of 68 genes related to immune, gut barrier, and neural function, in the colon tissue of germ-free mice after DSS administration, on control diet or HSD: C + D (control diet + DSS), HS + D (HSD + DSS). The heatmap was generated based on group average using Euclidean distance metric

We hypothesized that HSD exacerbates colitis through induction of gut dysbiosis as it decreases levels of beneficial bacteria, such as *Lactobacillus*, and decreases butyrate, a SCFA important for gut immune homeostasis and previously shown to be protective in colitis. To test this hypothesis, we administered 2% DSS to germ-free (GF) mice after 4 weeks of HSD. DSS administration induced colitis in GF mice (Fig. 5a, b), with increase in macroscopic scores and decreased colon length compared to control mice not exposed to DSS. However, there were no differences in macroscopic scores, colon length, MPO, or histology scores between GF mice on HSD and those on control diet after DSS-colitis (Fig. 5a–d). We assessed the expression of a group of relevant genes using a customized Nanostring gene expression codeset. DSS administration altered expression of 25 genes in GF mice on control diet (Additional file 4: Table S8) that were related to immune, barrier, and neural function such as Mapk1, Nfkb1, CD11c, and GABA B, confirming that the administration of DSS altered gut physiology, even in the absence of microbiota. However, out of the 68 tested genes, only 1 gene, Reg3ɣ, was found to be differentially expressed between HSD and control diet groups after DSS administration (log2 ratio = − 1.15; *p* = 0.032). These results suggest that the colonic microbiota is a key factor in exacerbation of colitis by HSD.

**Fig. 6:**
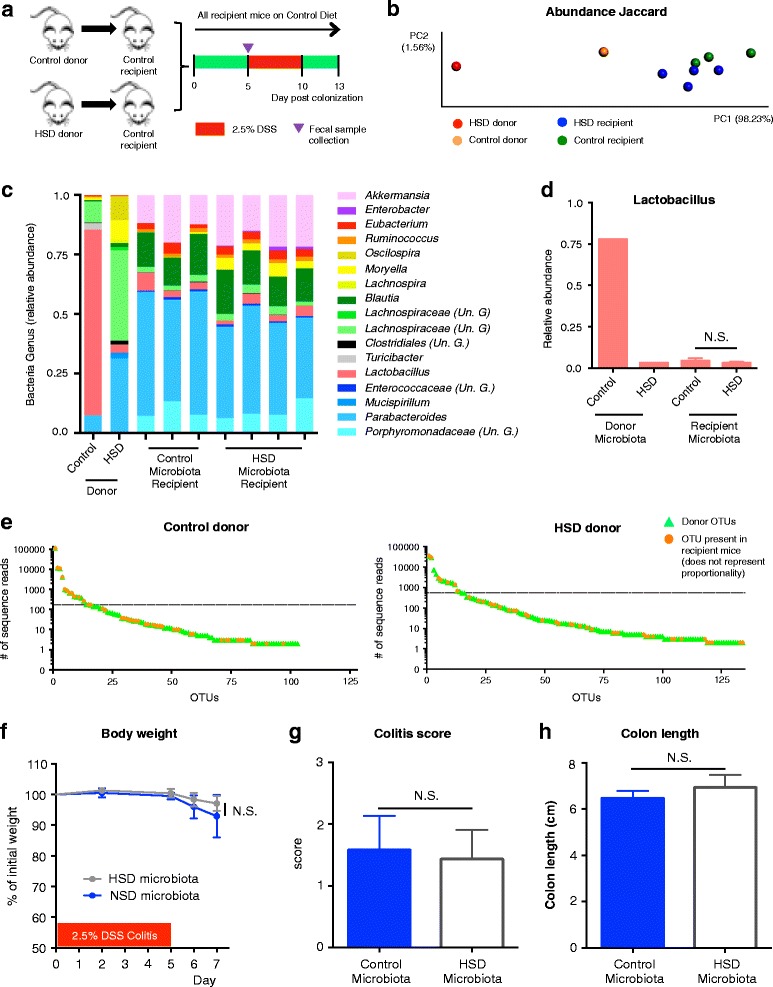
HSD-signature microbiota requires constant exposure to high salt diet for its maintenance. **a** Experimental design. **b** Principal coordinate analysis (PCoA) of abundance Jaccard index from fecal microbiota composition data. **c** Summary of the relative microbial genera present in 99.9% of the community. Un. G (Unclassified Genus). **d** Relative abundance of *Lactobacillus* genus. **e** Total read count of each OTU present in the indicated donor fecal microbiota (in green triangles). Orange dots indicate the OTUs that were present in recipient mice at day 5 post-colonization. The horizontal dashed line separates the 15 most abundant OTUs from the others. **f** Body weight during DSS administration. **g** Macroscopic scores of DSS colitis at endpoint. **h** Colon length at endpoint

To test whether HSD-associated microbiota alone can confer increased vulnerability to inflammatory insult, we transferred the microbiota from mice on HSD or control diet to GF mice. The recipient mice received control diet during the entire course of the experiment and were exposed to 1 cycle of DSS, starting at day 5 post-colonization (Fig. 6a). We observed no differences in colitis severity, including weight loss, colon length, and macroscopic scores between the two groups of mice (Fig. 6f–h). However, when analyzing the fecal microbiota profiles, we found that prior to DSS administration, both groups of recipient mice had similar microbial profiles, closer to control donor, based on abundance Jaccard index (Fig. 6b, c). Further analysis has confirmed that there were no differences between the microbiota genera of both recipient groups (Additional file 4: Table S10), including genera affected by HSD such as *Lactobacillus* (Fig. 6d). When analyzed in detail, the OTUs successfully transferred from control diet-microbiota accounted for 98.0% of the entire donor’s community, while the OTUs from HSD-microbiota accounted for 75.9% of the donor’s microbiota community (Table 2). From the 15 most abundant OTUs of each microbiota-type, that accounted for 99% of control diet microbiota and 95% of HSD microbiota, we verified that only 7 OTUs from HSD microbiota were detected in the recipient mice, while from control diet-microbiota 12 OTUs were present (Fig. 6e, Table 2). These results demonstrate that a significant proportion of the HSD OTUs did not successfully colonize the gut of recipient mice when these were fed control diet, suggesting that a constant exposure to high levels of NaCl in the diet is necessary to maintain the HSD signature microbiota profile. Taken together, similar colitis severity observed in the two microbiota-similar recipient groups supports our hypothesis that microbiota composition and function is a major determinant of colitis severity.

**Table 2:** OTU count analysis of microbiota transplant experiment

	# of OTUs		
	Total from donor	Successfully transferred to recipient	Transferred from 15 most abundant OTUs
CTL microbiota	103	43 (98.0%)^*^	12
HSD microbiota	134	43 (75.9%)^*^	7

^*^Coverage of the donor microbiota community based on read counts

## Discussion

In addition to host genetic make-up, environmental factors such as diet and microbiota composition have been shown to play a crucial role in the development of inflammatory diseases. Here, we show that high content of NaCl in diet modulates gut microbiota composition and function, namely, decreasing the relative abundance of *Lactobacillus* spp and levels of the SCFA butyrate, promoting a pro-inflammatory state in the gut. We demonstrate that the gut microbiota is a key mediator in the exacerbation of experimental colitis by HSD, supported by the fact that the detrimental effect of HSD was not observed in GF mice.

To understand the impact of HSD in the modulation of gut microbiota, we fed mice with a diet supplemented with 4% NaCl for 4 weeks and analyzed fecal microbiota dynamics during and after HSD. The NaCl concentration employed here simulates the concentration of a typical high salt diet [13, 40], which was shown to exacerbate the development of several immune disorders in mice [9, 10, 13]. Importantly, 4 weeks of 4% NaCl diet do not affect mouse blood pressure [9]. HSD altered gut microbiota composition and function, mainly decreasing colonic *Lactobacillus* spp. relative abundance and SCFA butyrate levels, two factors that have been implicated in the maintenance of a healthy gut physiology. This is in agreement with a very recently published study by Wilck et al. showing that high salt diet alters gut microbiota, mainly by decreasing levels of *Lactobacillus murinus*, leading to induction of T helper 17 cells [41] and that supplementation with *L. murinus* can abrogate the pro-inflammatory effect of HSD. In addition to butyrate, lactic acid also appeared to decrease after HSD, although not significantly. Interestingly, it has been shown that lactic acid is used by some butyrate-producing bacteria for the production of high concentrations of butyric acid [42], thus it is plausible that the decrease observed in *Lactobacillus* spp. is, at least in part, indirectly responsible for the significant decrease of butyric acid. Several species of *Lactobacillus* genus have been shown to play a role in host’s gut mucosal immune and barrier function [43]. A recent study showed that *Lactobacillus reuteri* ameliorates colitis in mice by increasing the colon mucus thickness [44]. Butyrate plays an essential role in gut homeostasis as it serves as the primary energy source for colonocytes and is important for maintaining tissue barrier function [45]. Moreover, it has been directly implicated in colonic Tregs’ differentiation [46, 47], intestinal macrophage function [48], and colonic inflammation downregulation [49]. Furthermore, a recent study has shown that butyrate deficiency is directly associated with colitis [50], while small clinical studies have shown that butyrate treatment improves colitis [51, 52].

We hypothesized that the changes in gut microbiota driven by HSD, in the absence of any additional inflammatory insult, would affect intestinal immune homeostasis. Our data revealed a strong impact of HSD on mucosal immunity, both in the colon and small intestine. Even though Na^+^ and Cl^−^ ions are thought to be absorbed in the small intestine, HSD had a stronger impact in the colon, when considering the number of affected genes (35 genes in the colon and 11 genes in the small intestine). This is in line with a recent study showing that HSD increased sodium luminal concentration in the colon but not in the small intestine [53], which may partially explain the different impact of HSD on small intestinal and colonic mucosal immunity.

Many of the altered genes were related to immune cell trafficking, cell viability, and signal transduction elements. Rac1 gene, which was increased by HSD, has been associated with ulcerative colitis in many studies. Several polymorphisms that lead to the increase of Rac1 expression have been shown in patients with UC [54] and Rac1 is the target of a commonly used IBD treatment azathioprine [55]. Moreover, mice with a Rac1 conditional deletion in neutrophils and macrophages are resistant to DSS-colitis, showing reduced neutrophil migration and reduced levels of IL1β and KC [54]. Furthermore, the genes Mapk3, Map2k1, Map2k6, Atf2, and Hras1, together with Rac1, were also integrated by IPA to be part of the upregulated “LPS-stimulated MAPK signaling” as well as part of other MAPK-related canonical pathways (Additional file 4: Table S7). This is of particular interest, not only because MAPK signaling pathway has been shown to be induced by NaCl [9, 10, 13], but also because microbiota-derived molecules such as LPS, flagellin, DNA, RNA, and others can lead to the activation of MAPK signaling pathways by selectively binding to various pattern recognition receptors on innate immune cells [56, 57]. In agreement with previous reports, we found that IL-17 signaling pathway was predicted to be affected by HSD in both colonic and small intestine tissues [9, 10, 13].

Surprisingly, we found that HSD downregulated the expression of multiple chemokines, cytokines, and its receptors in the colonic *lamina propria*. This was unexpected considering that previous studies have shown that NaCl induces macrophage chemotaxis in vitro [58] and that macrophages infiltrate the skin of rodents fed with HSD in response to the storage of Na^+^ in this organ, a mechanism important to maintain interstitial electrolyte and volume homeostasis [59]. However, several other studies have shown downregulation of the expression of chemokine receptors in neutrophils and other granulocytes, upon TLR engagement in a p38-MAPK signaling-dependent manner, with cells remaining partially functionally competent; a potential mechanism regulating cell positioning and activation [60, 61]. Considering that HSD induced changes in colonic microbiota and upregulation of MAPK signaling pathways in the colon, it is conceivable that the observed downregulation of chemokine receptors and other molecules is a result of mucosal granulocytes TLR engagement. Downregulation of cytokines and chemokines could compromise the colonic immune barrier and promote a pro-inflammatory response or impaired resolution of inflammation after the intestinal injury induced by DSS and DNBS.

To investigate the effect of HSD-induced alterations in mucosal immune function and gut microbiota, we used two different models of experimental colitis. We confirmed that HSD exacerbates colitis in mice [13] and showed enhanced colonic barrier disruption after DSS in mice fed with HSD. In fact, Ocln and ZO-1, two components of the tight junction (TJ) structure, were significantly downregulated, as well as Muc2, a protein secreted by goblet cells (GC) and essential for the structure of the mucus layer in the intestine, and Tff3, also mostly expressed by GC, with a key role in mucus layer stability and mucosal repair [62]. On the other hand, Mmp9 and Cnlp were upregulated in HSD mice after DSS-colitis. Even though Cnlp has been proposed to have a protective role [63], the level of this peptide has been shown to be elevated in DSS colitis and patients with ulcerative colitis [64, 65]. Mmp9 is markedly elevated in intestinal tissues of IBD patients, and it has been proposed to be a biomarker of disease activity [66–68]. Mmp9 is also elevated in DSS colitis and has been shown to contribute to the severity of colitis by increasing intestinal epithelial TJ permeability [68]. Cnlp and Mmp9 are highly expressed by active neutrophils; therefore, their upregulation could also reflect the increased infiltration of these cells in the inflamed colonic tissue of HSD mice after DSS administration. We reproduced the colitis results in a different mouse strain and showed that HSD leads to a similar exacerbation of DSS colitis in NIH Swiss as in C57BL/6 mice, suggesting that HSD-mediated exacerbation of experimental colitis is independent of mouse genotype. Further supporting our results, several studies have recently reported increased severity of colonic inflammation not only in models of chemically induced colitis [13, 69], but also in IL-10 KO mice, which spontaneously develop colitis, and in *Salmonella typhimurium* infected mice, while on HSD [53].

In order to test whether gut microbiota plays a role in the HSD-dependent exacerbation of gut injury, we induced DSS colitis in GF mice. While DDS induced colitis in both groups of germ-free mice, there was no worsening of the colonic inflammation in mice fed a HSD. This suggests that the increased susceptibility to colitis induced by a HSD is critically dependent on the presence of the microbiota. In subsequent experiments, we found no difference in colitis severity when transferring gut microbiota from SPF mice on a HSD into germ-free recipients. Although seemingly counterintuitive, the results are in accordance with our previous data as we found that at the beginning of DSS administration (5 days post-colonization), the microbiota of all recipient mice had converged to a similar profile, regardless of the donor microbiota. This is in sharp contrast with our SPF mice experiment, where we observed a marked dysbiosis prior to commencing DSS and subsequently worse colitis in mice previously on high salt diet. In our transfer experiment, most of the abundant OTUs from the control diet donor were transferred to the recipient mice while only a minority of abundant HSD donor OTUs were detected in the recipient mice. These differences in microbiota profiles could be partially attributed to technical issues, including freezing and thawing, and exposure to oxygen, as we did not use fresh cecal content samples for colonizations. However, we believe that the major factor accounting for this difference was the fact that recipient mice were given control diet, which did not allow establishment of HSD microbiota profiles. Thus, the microbiota transfer experiment supports the notion that HSD-induced dysbiosis, and its altered metabolic activity, is a major determinant of the sensitivity to inflammatory insult in this model and that a constant exposure to HSD is necessary to maintain the “pro-inflammatory” microbiota profile.

Even though it has been previously shown that NaCl can directly affect immune cells in vitro, we hypothesize that these direct effects do not play a major role in our in vivo model. Taking into consideration that (i) dysbiosis, including the depletion of *Lactobacillus* and butyrate, is known to affect mucosal immune homeostasis, and that (ii) the mucosal milieu in the colon, which harbors the highest microbial density along the GI tract, was more affected by HSD than small intestine, and that (iii) multiple immune pathways affected by HSD are strongly modulated by microbial products, we hypothesize that changes in the immune system are a consequence of the disruption of the gut microbiota by HSD. This notion is further supported by the lack of changes in colonic mucosal immune milieu in germ-free mice on HSD (Additional file 4: Table S9).

Taken together, this study provides an example of how environmental factors, such as diet, play a key role in shaping gut microbial communities that influence host physiology and predisposition to disease.

## Conclusion

We have demonstrated that consumption of a diet with high salt content results in alteration of gut microbiota composition and function that may be pro-inflammatory by virtue of the loss of *Lactobacillus* spp. and reduced production of the protective SCFA butyrate. Our findings indicate that the maintenance of the pro-inflammatory microbiota is dependent on a continued high salt intake and is associated with changes in gut immune homeostasis, predisposing mice to the development of a more severe form of experimental colitis. Our study suggests that high concentrations of dietary salt, similar to those found in the western diet, contribute to the increased prevalence of chronic inflammatory disorders through microbiota-dependent mechanisms. This finding has implications for the management of patients with IBD and other inflammatory disorders and should be further investigated as it provides an easy candidate for dietary therapeutic interventions.

## Additional files

Additional file 1: 16S metadata and experiment raw data. (XLS 219 kb)

Additional file 2: Supplementary methods. (PDF 133 kb)

Additional file 3: Nanostring raw counts. (XLS 108 kb)

Additional file 4: Supplementary figures and tables. (PDF 1.22 mb)
