# Supplementary material for: High salt diet exacerbates colitis in mice by decreasing Lactobacillus levels and butyrate production
Source: Microbiome. 2018 Mar 22;6:57. doi: 10.1186/s40168-018-0433-4 (PMC5865374; doi:10.1186/s40168-018-0433-4)
Supplement: Supplementary file 2 — Supplementary methods. (PDF 133 kb) [file 40168_2018_433_MOESM2_ESM.pdf]

Supplementary Methods

Macroscopic evaluation of DSS colitis in mice

**Presence of pellets in proximal colon**	yes	0
	no	0.5
**Presence of pellets in distal colon**	yes	0
	no	0.5
**Pellet consistency**	normal	0
	soft	0.5
	diarrhea	1
**Blood in anus**	yes	0.5
	no	0
**Blood in cecum**	yes	0.5
	no	0
**Blood in colon**	yes	0.5
	no	0
**Bloody stool**	yes	0.5
	no	0
**Pale and rigid distal colon**	yes	0.5
	no	0

Macroscopic evaluation of DNBS colitis in mice

**Ulcer**	# of ulcers and size (cm)
**Diarrhea**	(0-3)
**Adhesions**	(0-3)
**Thickness**	(0-3)
**Hyperemia**	(0-3)
**Inflammation**	(0-3)

Ulcer scoring:

2 points for each ulcer < than 0.5cm

3 points for each ulcer 0.5cm < x < 1cm

4 points for each ulcer => than 1cm

Histology scores

**Loss of architecture**	(0-3)
**Cellular infiltrate**	(0-3)
**Goblet cell depletion**	(0-1)
**Ulcers**	(0-1)
**Edema**	(0-1)
**Muscle thickening**	(0-2)
**Crypt abscess**	(0-1)

Primer's list

All primers used for Q-PCR analyses were QuantiTech Primer Assay primers (Qiagen). Mm_Csf2 (Ref: 249900), Mm_Alox15_1 (Ref: 249900), Mm_Atf2_1 (Ref: 249900), Mm_Rac1_1 (Ref: 249900). As reference gene we used the TATA-box biding protein (Tbp) as previously described[1].

1. Eissa N, Hussein H, Wang H, Rabbi MF, Bernstein CN, Ghia J-E. Stability of Reference Genes for Messenger RNA Quantification by Real-Time PCR in Mouse Dextran Sodium Sulfate Experimental Colitis. PLoS One. 2016;11(5).

R Code for Heatmap

```
row.names(matrix_data) <- PICRUSt$`KEGG Pathways`
matrix_data <- data.matrix(PICRUSt[,2:ncol(PICRUSt)])
my_palette <- colorRampPalette(c("blue", "white", "red"))(n=3*50-1)
shades <- c(seq(-3,-0.25,length=50),seq(-0.24,0.25,length=50),seq(0.26,3,length=50))
heatmap.2(matrix_data, margins = c(10,20), breaks = shades, trace = "none", keysize=1, key.par = list(cex=0.5), colsep = c(5,11,16), rowsep = c(1:20), sepcolor="white", sepwidth=c(0.025,0.05), scale = "row", Rowv = FALSE, Colv = FALSE, dendrogram = "none", cexCol = 0.5, cexRow = 0.5, main = "KEGG Pathways 2", col = my_palette)
```

Nanostring custom genes panel

Official Symbol	Name	Accession #
5HT	Serotonin	NM_010484.2
BDNF	Brain-derived neurotrophic factor	NM_007540.4
C3	Complement component 3	NM_009778.2
CALB	Calbindin 1	NM_009788.4
CALRET	Calreticulin	NM_007591.3
Casp8	Caspase 8	NM_009812.2
CCR2	C-C chemokine receptor type 2	NM_009915.2
CCR6	C-C chemokine receptor type 6	NM_001190333.1
CD11b	Integrin subunit alpha M	NM_001082960.1
CD11C	Integrin subunit alpha X	NM_021334.2
Cd86	CD86	NM_019388.3
Cdh1	E-cadherin	NM_009864.2
CGRP	Calcitonin gene-related peptide	NM_007587.2
CHAT	Choline acetyltransferase	NM_009891.2
Cldn1	Claudin 1	NM_016674.3
Cldn2	Claudin 2	NM_016675.3
Cnlp	Cathelin-related antimicrobial peptide (CRAMP)	NM_009921.2
Cxcr3	C-X-C motif chemokine receptor 3	NM_009910.2
Cxcr4	C-X-C motif chemokine receptor 4	NM_009911.3
Defcr1	Defensin-related cryptdin peptide-1	NM_010031.2
F2rl1	Protease activated receptor 2 (PAR2)	NM_007974.4
GABA A	Gamma-aminobutyric acid A	NM_010252.4
GABAB	Gamma-aminobutyric acid B	NM_019439.3
GATA-3	GATA Binding Protein 3	NM_008091.3
GDNF	Glial Cell Derived Neurotrophic Factor	NM_010275.2
GFAP	Glial Fibrillary Acidic Protein	NM_001131020.1
Gpr44	Prostaglandin D2 Receptor 2	NM_009962.2
Hif1a	Hypoxia Inducible Factor 1 Alpha Subunit	NM_010431.2
HMGB1	High Mobility Group Box 1	NM_010439.3
I-FABP	Fatty acid-binding protein 2	NM_007980.2
Il17a	Interleukin-17A	NM_010552.3
Il22ra2	Interleukin 22 Receptor Subunit Alpha 2	NM_178258.5
Il23r	Interleukin 23 receptor	NM_144548.1
Lyz1	Lysozyme 1	NM_013590.3
Mapk1	Mitogen-Activated Protein Kinase 1	NM_011949.3
MMP9	Matrix Metallopeptidase 9	NM_013599.2
Muc2	Mucin 2	NM_023566.2
Myd88	Myeloid Differentiation Primary Response 88	NM_010851.2
Mylk3	myosin light chain kinase 3	NM_175441.5
NFAT	Nuclear factor of activated T-cells	NM_010901.2
Nfkb1	Nuclear factor NF-kappa-B p105 subunit	NM_008689.2
Nod2	Nucleotide-binding oligomerization domain-containing protein 2	NM_145857.2
NOS	Nitric Oxide Synthase 1	NM_008712.2
NPY	Neuro peptide Y	NM_023456.2
NR2B	Glutamate Ionotropic Receptor NMDA Type Subunit 2B	NM_008171.3
NR2D	Glutamate Ionotropic Receptor NMDA Type Subunit 2D	NM_008172.2
Ocln	Occludin	NM_008756.2
P75	Nerve Growth Factor Receptor	NM_033217.3
PGP9.5	Ubiquitin C-Terminal Hydrolase L1	NM_011670.2
Pparg	PPAR-gamma, peroxisome proliferator activated receptor gamma	NM_011146.1
Rae1	Ribonucleic Acid Export 1	NM_175112.5
Reg3g	Regenerating Family Member 3 Gamma	NM_011260.1
S100BETA	S100 Calcium Binding Protein B	NM_009115.3
SEMAPHORIN	Semaphorin	NM_011352.2
SOX10	SRY-Box 10	NM_011437.1
SUBP	Substance P	XM_006505028.1
T-bet	T-box 21	NM_019507.1
TAAR1	Trace amine associated receptor 1	NM_053205.1
TAAR4	Trace amine associated receptor 4	NM_001008499.1
Tff3	Trefoil factor 3	NM_011575.2
TGR5	G-protein coupled bile acid receptor 1	NM_174985.1
Tjp1	Tight junction Protein 1 (ZO-1)	NM_009386.1
Tlr2	Toll-like receptor 2	NM_011905.2
Tlr4	Toll-like receptor 4	NM_021297.2
Tlr5	Toll-like receptor 5	NM_016928.2
Tollip	Toll Interacting Protein	NM_023764.3
trpv1	transient receptor potential cation channel, subfamily V, member 1	NM_001001445.1
VIP	Vasoactive Intestinal Peptide	NM_011702.2
