# Supplementary material for: High salt diet exacerbates colitis in mice by decreasing Lactobacillus levels and butyrate production
Source: Microbiome. 2018 Mar 22;6:57. doi: 10.1186/s40168-018-0433-4 (PMC5865374; doi:10.1186/s40168-018-0433-4)
Supplement: Supplementary file 4 — Supplementary figures and tables. (PDF 1.22 mb) [file 40168_2018_433_MOESM4_ESM.pdf]

Figure S1

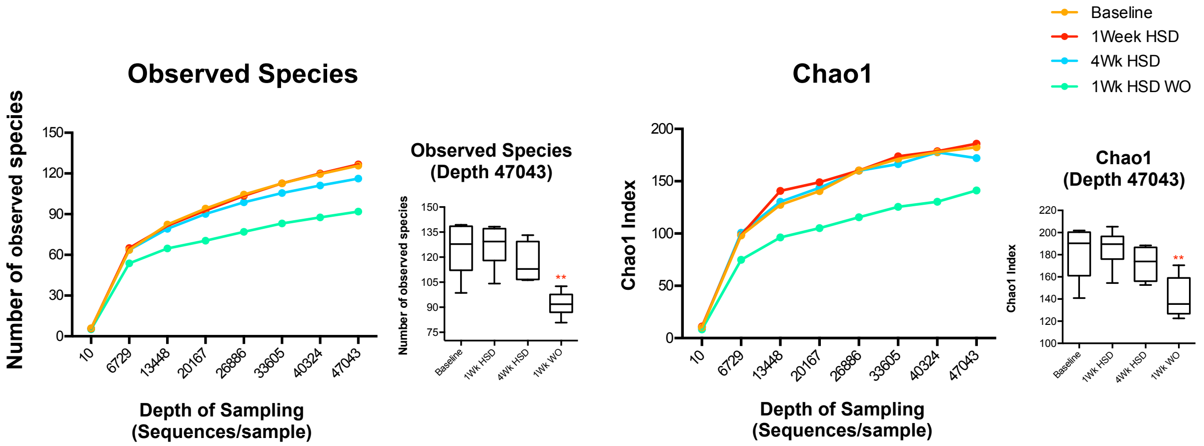

Supplementary Figure 1. HSD affects richness and diversity of fecal microbiota. Observed species and Chao1 diversity index of fecal microbiota at the indicated time points after introduction of HSD.

Figure S2

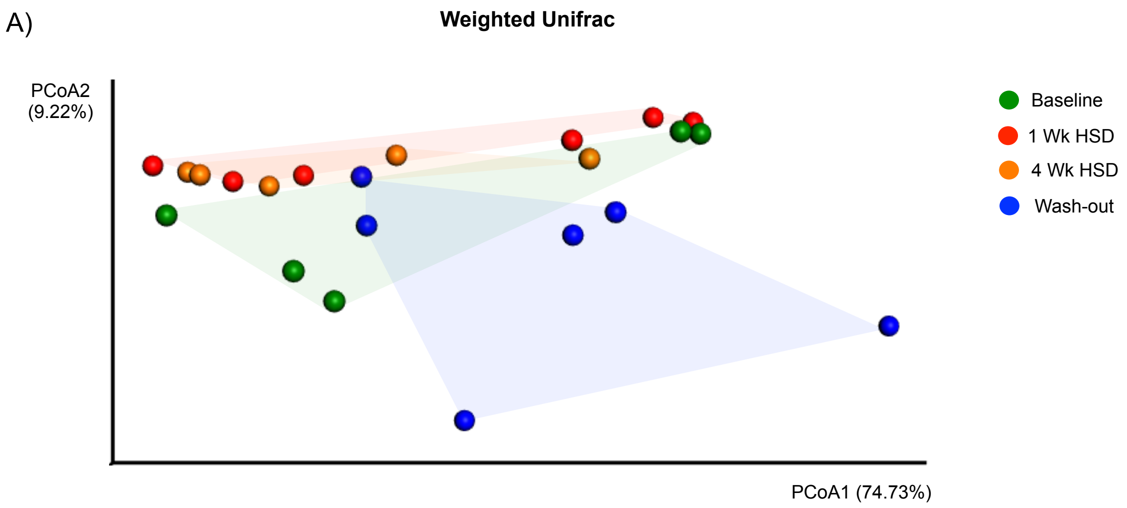

Supplementary Figure 2. Weighted Unifrac distance metrics of gut microbiota of mice fed with HSD. PCoA plot of weighted unifrac metric of fecal microbiota composition over time exposed to HSD.

Figure S3

A)

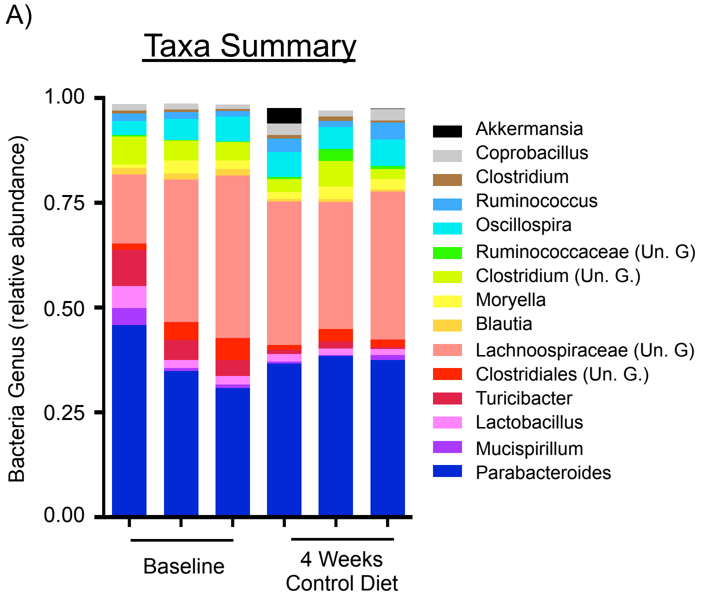

B)

Test-Statistic	P-Value	FDR_P	TAXONOMY: GENERA
-7.639	0.002	0.028	*Blautia*
6.757	0.003	0.028	*Bifidobacterium*
4.530	0.011	0.078	*Bacteria (Un. P)*
-3.080	0.037	0.203	*Turicibacter*
2.587	0.061	0.268	*Erysipelotrichaceae (Un. G)*
2.361	0.078	0.284	*Coprobacillus*
2.079	0.106	0.333	*Lachnospiraceae (Un. G)*
1.712	0.162	0.405	*Ruminococcus*
1.670	0.170	0.405	*Ruminococcus*
1.571	0.191	0.405	*Ruminococcaceae (Un. G)*
-1.488	0.211	0.405	*Lactobacillus*
-1.448	0.221	0.405	*Clostridiales (Un. F)*
1.200	0.296	0.484	*Oscillospira*
1.131	0.321	0.484	*Clostridium* (from *Erysipelotrichaceae* Family)
1.082	0.340	0.484	*Akkermansia*
-1.019	0.366	0.484	*Clostridium*
-1.001	0.374	0.484	*Mucispirillum*
-0.536	0.620	0.708	*Anaerotruncus*
0.525	0.627	0.708	*Moryella*
0.499	0.644	0.708	*Lachnospiraceae (Un. G)*
-0.187	0.861	0.902	*Clostridium* (From *Clostridiaceae* Family)
0.083	0.938	0.938	*Parabacteroides*

C)

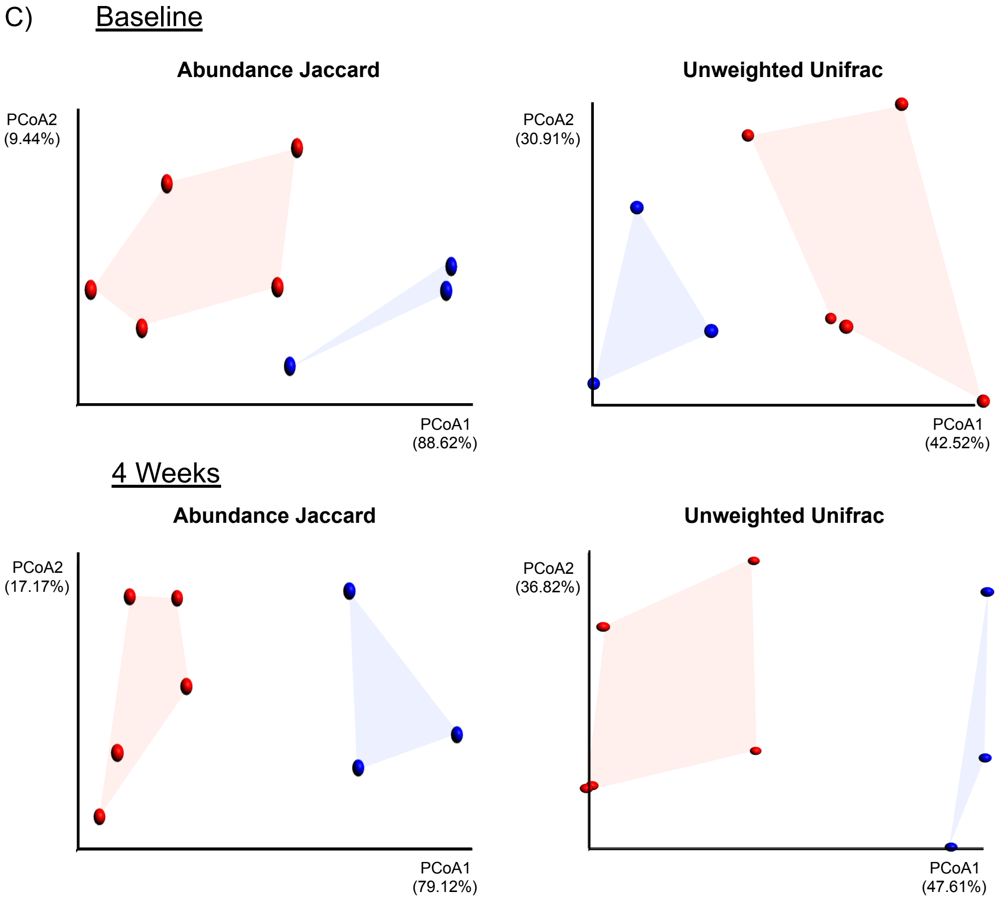

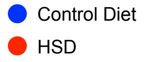

	Baseline	
	Ab. Jaccard	Unw. Unifrac
Anosim R	0.713	0.426
p-value	0.039	0.035

	4 Weeks	
	Ab. Jaccard	Unw. Unifrac
Anosim R	0.928	0.744
p-value	0.018	0.024

Supplementary Figure 3. Gut microbiota is stable in mice on control diet during week 8-12 A: Summary of the relative abundance of microbial genera present in 99.9% of the community before and after 4 weeks on control diet. (Un. G: Unclassified Genus) B: Summary of the t-test statistical analysis of microbiota genera before and after 4 weeks on control diet. Highlighted in red are the taxa that were shown to correlate with HSD. C: PCoA plots of abundance Jaccard index (left panels) and unweighted unifrac metric (right panels) of fecal microbiota composition from mice on control and high salt diet, at the beginning of the experiment (Baseline) and 4 Weeks of diet. Anosim statistical analysis of respective distance metrics.

Figure S4

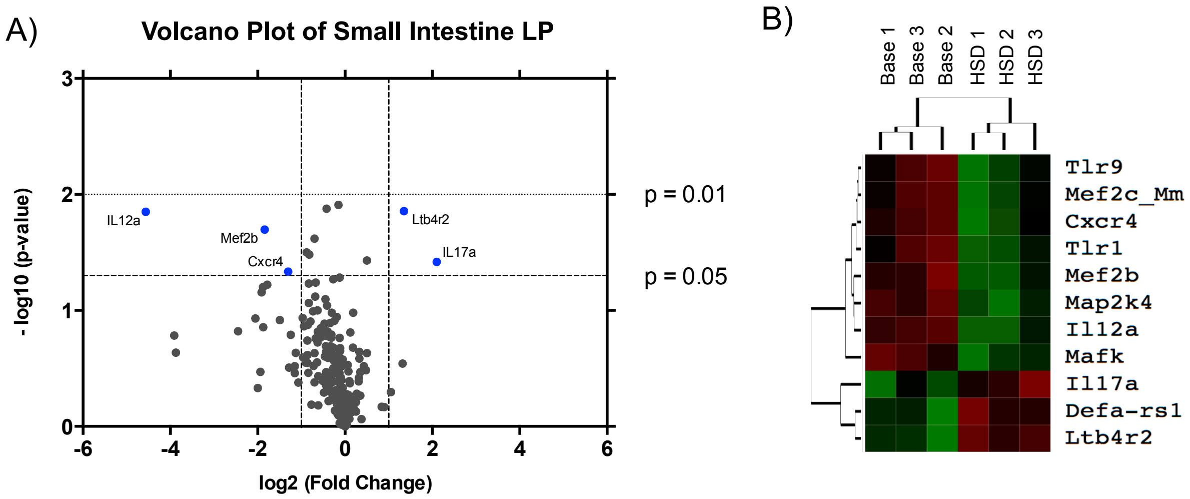

Supplementary Figure 4. HSD modulates immune expression in small intestine *lamina propria*. A: Volcano plot of gene expression in small intestine *lamina propria* total cell extract of mice receiving HSD or control diet for 4 weeks, n=9 mice/group. Blue dots: genes with $p < 0.05$, fold change > 2. B: Heatmap of immune genes that changed with HSD ($p<0.05$). The heatmap was generated based on group average, using Euclidean distance metric.

Figure S5

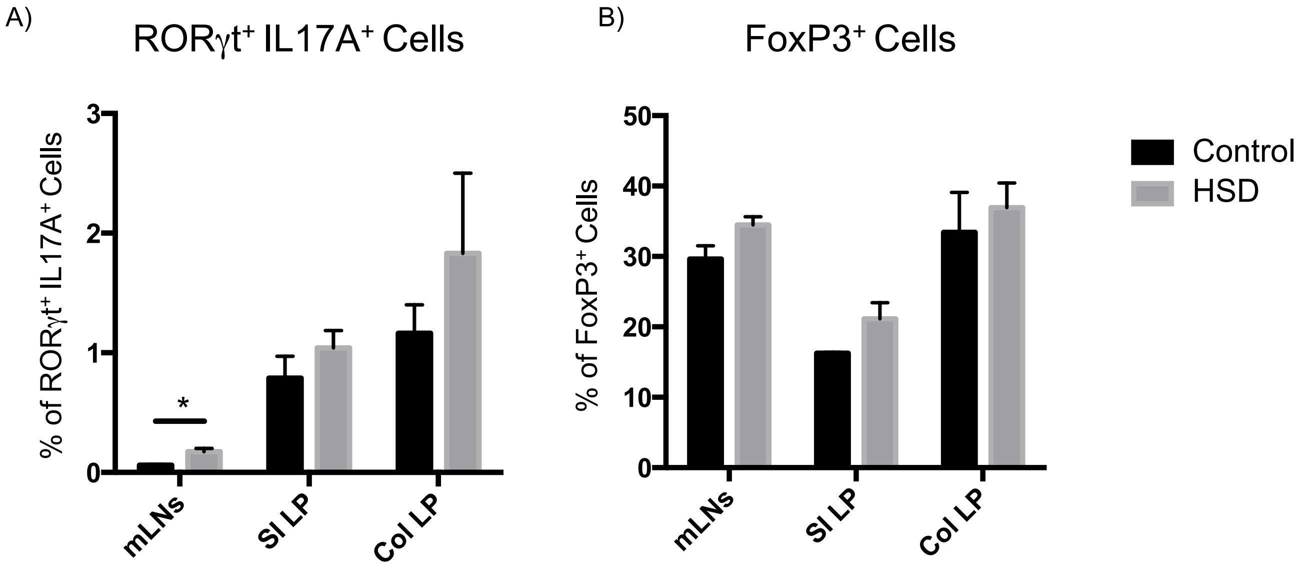

Supplementary Figure 5. HSD increases RORgt$^+$ IL17$^+$ Cells in mLN but it doesn't affect FoxP3 cells. Total cell extract of mesenteric lymph nodes (mLNs), small intestine *lamina propria* (SI LP) and colonic *lamina propria* (Col LP) was performed on mice fed HSD for 4 weeks (and control diet). Cells were analyzed with flow cytometry. Indicated % are from viable cells gated on TCRb$^+$ CD4$^+$. This is a representative experiment of 3 other different independent experiments. Values are presented as means ± SEM; n = 6-9 mice/group. * ($p<0.05$)

Figure S6

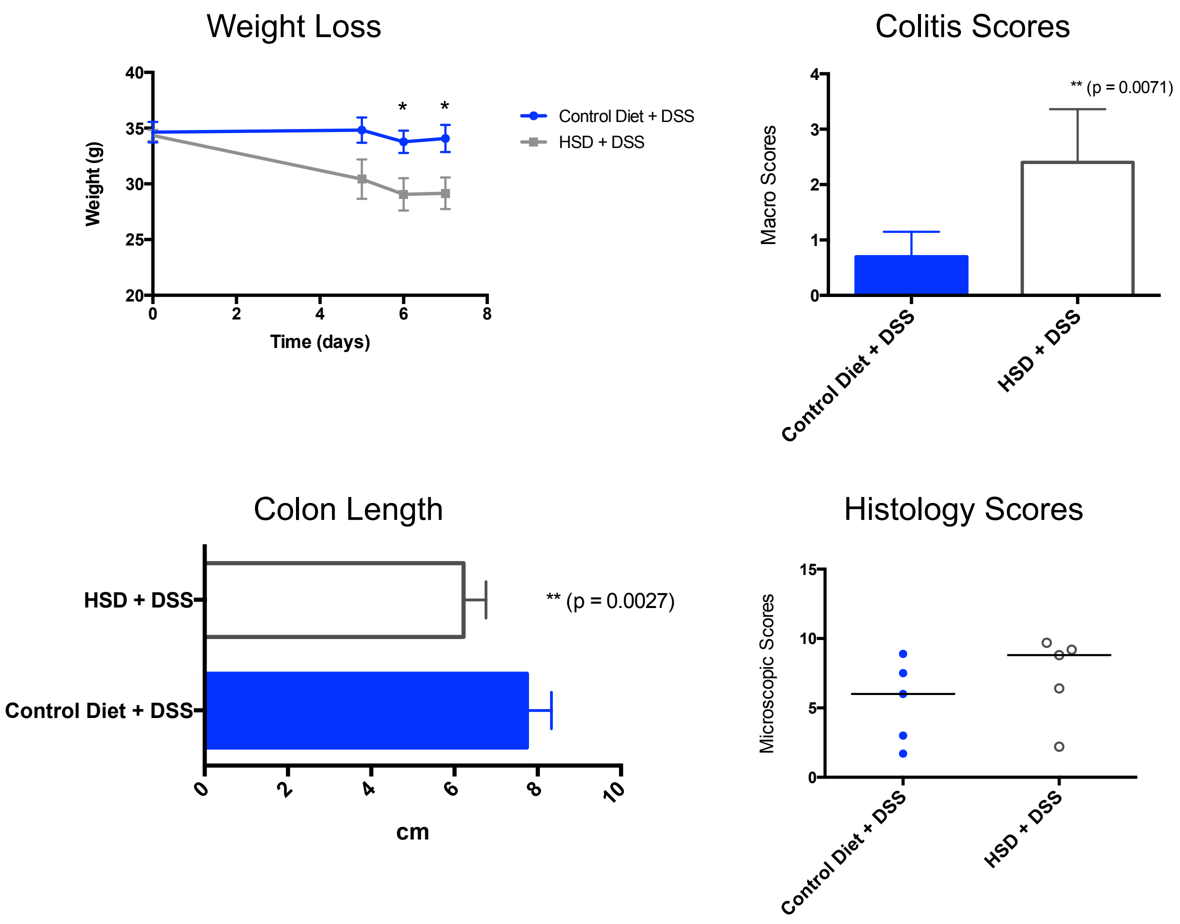

Supplementary Figure 6. HSD increases susceptibility to DSS colitis in NIH Swiss mice. Mice, previously fed HSD or control diet, were exposed to 3.5% DSS in drinking water during 5 days. DSS (Control diet, DSS in water), HSD+DSS (High salt diet, DSS in water). A: Body weight changes during DSS treatment B: Clinical assessment of DSS colitis at endpoint (Day 7). C: Colon length at endpoint. D: Histology score of colonic sections. Values are presented as means ± SEM; n = 5 mice/group. * ($p<0.05$) ** ($p<0.01$).

Table S1

Table S1 - Fecal microbiota genera that significantly correlates with HSD

Taxonomy (Genus)	Spearman r	p-value	q (FDR)
Lactobacillus	-0.703	1.39E-04	0.003
Lachnospiraceae (Un. Genus)	0.614	0.002	0.017
Clostridiales (Un. Genus)	-0.605	0.002	0.017
Oscillospira	0.548	0.007	0.042

Table S2

Table S2 - Fecal microbiota OTU that significantly correlates with HSD

Taxonomy (OTUs)	Spearman r	p-value	q (FDR)
OTU3: *g__Lactobacillus*	-0.707	1.22E-04	0.005
OTU9: *o__Clostridiales*	-0.684	2.62E-04	0.005
OTU25: *f__Lachnospiraceae*	0.609	0.002	0.027
OTU10: *g__Oscillospira*	0.482	0.022	0.214
OTU24: *f__Lachnospiraceae*	0.454	0.033	0.214
OTU16: *f__Lachnospiraceae*	-0.454	0.033	0.214
OTU2: *f__Lachnospiraceae*	0.427	0.047	0.260

Table S3

Table S3 - List of the genes that are significantly altered by HSD in colonic lamina propria

Gene Code	Gene Name	Accession #	log2(ratio)	P value	Type
Cytokines, Chemokine and Receptors					
Ccl3	*C-C motif Chemokine Ligand 3*	NM_007482.3	-1.42	0.0091	Chemokine
Ccl4	*C-C motif Chemokine Ligand 4*	NM_144938.2	-1.45	0.0182	Chemokine
Cxcl10	*C-X-C motif Chemokine Ligand 10*	NM_009777.2	-0.9	0.017	Chemokine
Cxcl2	*C-X-C motif Chemokine Ligand 2*	NM_016960.1	-2.07	0.039	Chemokine
Ccr7	*C-C motif Chemokine Receptor 7*	NM_013653.1	-1.9	0.047	Chemokine Receptor
Cxcr4	*C-X-C motif Chemokine Receptor 4*	XM_356827.6	-1.5	0.0259	Chemokine Receptor
Csf2	*Colony Stimulating Factor*	NM_007465.2	-1.73	0.0157	Cytokine
Ifi27l2a	*Interferon Alpha Inducible Protein 27 Like 2*	NM_009744.3	-1.13	0.0101	Cytokine
Il1b	*Interleukin 1 Beta*	NM_013654.2	-1.39	0.0476	Cytokine
Il7	*Interleukin 7*	NM_009662.2	0.47	0.0033	Cytokine
Tnfsf14	*Tumor Necrosis Factor Superfamily Member 14*	NM_023143.3	-1.39	0.0181	Cytokine
Il1rap	*Interleukin-1 Receptor Accessory Protein*	NM_007440.4	0.19	0.0005	Cytokine receptor
Cysltr2	*Cysteinyl Leukotriene Receptor 2*	NM_133882.2	-1.11	0.0287	Leukotrienes Receptor
Ltb4r1	*Leukotriene B4 Receptor 1*	NM_019577.4	-0.78	0.043	Leukotrienes Receptor
Transcription Factors and Regulators					
Atf2	*Activating Transcription Factor 2*	NM_146148.1	0.27	0.0265	Transcription Factor
Cebpb	*CCAAT/Enhancer Binding Protein Beta*	NM_007572.2	-0.87	0.0165	Transcription Factor
Irf5	*Interferon Regulatory Factor 5*	NM_011888.2	-0.71	0.0343	Transcription Factor
Mef2c_Mm	*Myocyte-specific Enhancer Factor 2C*	NM_009778.2	-0.67	0.021	Transcription Factor
Myc	*V-Myc Avian Myelocytomatosis Viral Oncogene Homolog*	NM_011332.2	-0.39	0.0316	Transcription Factor
Keap1	*Kelch-like ECH-associated Protein 1*	NM_013652.1	0.35	0.045	Regulatory Element
Signal Transduction Elements					
Ptgs2	*Prostaglandin-endoperoxide Sunthase 2 (aka COX-2)*	NM_011330.3	-1.67	0.0307	Signal Transduction Enzyme
Map2k1	*Mitogen-Activated Protein Kinase Kinase 1*	NM_009704.3	0.59	0.0061	Signal Transduction Kinsases
Map2k6	*Mitogen-Activated Protein Kinase Kinase 6*	NM_016704.2	0.41	0.0219	Signal Transduction Kinsases
Mapk3	*Mitogen-Activated Protein Kinase 3*	NM_009743.4	0.52	0.01	Signal Transduction Kinsases
Ptger3	*Prostaglandin E Receptor 3*	NM_009779.2	1.35	0.021	Signal Transduction Receptor
Gnb1	*G-Protein Subunit Beta 1*	NM_009660.3	0.25	0.0015	Signaling Transduction
H2-Eb1	*Histocompatibility 2, class II antigen E Beta*	NM_011124.4	-0.77	0.0394	Signaling Transduction
Hras1	*GTPase Hras (aka Transforming Protein p21)*	NM_013484.2	-2.41	0.0192	Signaling Transduction
Rac1	*Ras-related C3 botulinum toxin substrate 1*	NM_007425.2	0.41	0.0001	Signaling Transduction
Tyrobp	*TYRO Protein Tyrosine Kinase Binding Protein*	NM_013485.1	-1.39	0.03	Signaling Transduction
Other					
Alox15	*Arachidonate 15-lipoxygenase*	NM_001025093.1	-3.27	0.0098	Lipid Oxidation Enzyme
Alox5	*Arachidonate 5-lipoxygenase*	NM_009137.2	-1.84	0.0411	Lipid Oxidation Enzyme
Arg1	*Arginase1*	NM_011413.2	-0.6	0.0216	Enzyme
Itgb2	*Integrin Subunit Beta 2 (aka CD18)*	NM_011337.1	-0.77	0.0444	Integrin
Cfb	*Complement Factor B*	NM_011333.3	0.41	0.0346	Complement Factor

*From IPA analysis

Table S4

Table S4 - Most significant canonical pathways altered by HSD in colonic lamina propria

**Canonical Pathways**	**Genes involved**	**p-value**	**Ratio**	**z-score**
**Colon**				
Role of Macrophages, Fibroblasts and Endothelial Cells in Rheumatoid Arthritis	*MAP2K6,MYC,MAPK3,RAC1,HRAS,IL1B,CEBPB,CSF2,MAP2K1,IL1RAP,IL7,ATF2*	1.07E-12	4.03E-02	NaN
Role of IL-17F in Allergic Inflammatory Airway Diseases	*CXCL10,CCL4,MAPK3,IL1B,CSF2,MAP2K1,ATF2*	3.22E-12	1.71E-01	0
IL-17 Signaling	*CXCL10,MAP2K6,MAPK3,HRAS,CEBPB,PTGS2,MAP2K1,ATF2*	1.05E-11	9.52E-02	NaN
Communication between Innate and Adaptive Immune Cells	*CXCL10,CCL4,CCL3L3,IL1B,CSF2,HLA-DRB5,CCR7*	6.07E-10	8.33E-02	NaN
Acute Phase Response Signaling	*MAP2K6,MAPK3,CFB,HRAS,IL1B,CEBPB,MAP2K1,IL1RAP*	1.34E-09	5.23E-02	0.707
Eicosanoid Signaling	*ALOX15,LTB4R,PTGER3,PTGS2,ALOX5,CYSLTR2*	4.57E-09	9.68E-02	NaN
IL-6 Signaling	*MAP2K6,MAPK3,HRAS,IL1B,CEBPB,MAP2K1,IL1RAP*	8.45E-09	5.74E-02	0.378
LPS-stimulated MAPK Signaling	*MAP2K6,MAPK3,RAC1,HRAS,MAP2K1,ATF2*	3.36E-08	6.98E-02	1.633
p38 MAPK Signaling	*MAP2K6,MYC,IL1B,MEF2C,IL1RAP,ATF2*	1.48E-07	5.45E-02	0

*From IPA analysis

Table S5

Table S5 - Most significant functions or diseases altered by HSD in colonic lamina propria

	Funtion Or Disease	Genes involved	p-value	Activation
	**Colon**			
Top 5 (Lowest P-values)	adhesion of immune cells	*ALOX15,CCL3L3,CCL4,CCR7,CSF2,CXCL10,CXCL3,CXCR4,HRAS,IL1B,IL7,ITGB2,LTB4R,MAP2K1,PTGS2,RAC1*	3.18E-18	-2.268
	cellular homeostasis	*ARG1,CCL3L3,CCL4,CCR7,CSF2,CXCL10,CXCL3,CXCR4,HRAS,IL1B,IL7,IRF5,ITGB2,LTB4R,MAP2K1,MAPK3,MEF2C,MYC,PTGS2,RAC1,TYROBP*	8.01E-17	-1.409
	leukocyte migration	*ALOX15,ALOX5,CCL3L3,CCL4,CCR7,CSF2,CXCL10,CXCL3,CXCR4,HRAS,IL1B,IL7,IRF5,ITGB2,LTB4R,MYC,PTGS2,RAC1,TNFSF14,TYROBP*	9.04E-17	-3.291
	cell movement of leukocytes	*ALOX5,CCL3L3,CCL4,CCR7,CSF2,CXCL10,CXCL3,CXCR4,HRAS,IL1B,IL7,ITGB2,LTB4R,MYC,PTGS2,RAC1,TNFSF14,TYROBP*	1.64E-15	-3.017
	inflammatory response	*ALOX5,CCL3L3,CCL4,CCR7,CEBPB,CSF2,CXCL10,CXCL3,CXCR4,IL1B,ITGB2,LTB4R,PTGS2,RAC1,TNFSF14,TYROBP*	4.47E-15	-3.311
Bottom 5 z-score	Inflammatory Response	*ALOX5,CCL3L3,CCL4,CCR7,CEBPB,CSF2,CXCL10,CXCL3,CXCR4,IL1B,ITGB2,LTB4R,PTGS2,RAC1,TNFSF14,TYROBP*	4.47E-15	-3.3
	Leukocyte Migration	*ALOX15,ALOX5,CCL3L3,CCL4,CCR7,CSF2,CXCL10,CXCL3,CXCR4,HRAS,IL1B,IL7,IRF5,ITGB2,LTB4R,MYC,PTGS2,RAC1,TNFSF14,TYROBP*	9.04E-17	-3.29
	Cell Movement of myeloid Cells	*ALOX5,CCL3L3,CCL4,CCR7,CSF2,CXCL10,CXCL3,CXCR4,HRAS,IL1B,ITGB2,LTB4R,PTGS2,RAC1,TYROBP*	2.69E-14	-3.26
	Migration of Cells	*ALOX15,ALOX5,CCL3L3,CCL4,CCR7,CSF2,CXCL10,CXCL3,CXCR4,HRAS,IL1B,IL7,IRF5,ITGB2,LTB4R,MAP2K1,MYC,PTGS2,RAC1,TNFSF14,TYROBP*	8.76E-15	-3.2
	Cell Movement of phagocytes	*ALOX5,CCL3L3,CCL4,CCR7,CSF2,CXCL10,CXCL3,CXCR4,HRAS,IL1B,ITGB2,LTB4R,PTGS2,RAC1,TYROBP*	3.99E-14	-3.01
Top 5 z-score	Cell Viability of lymphocytes	*IL7,MAP2K1,MAPK3,MYC*	5.98E-04	1.925
	Cell Viability of mononuclear leukocytes	*CSF2,IL7,MAP2K1,MAPK3,MYC*	5.01E-05	1.478
	Maturation of Lymphocytes	*CSF2,IL7,MAPK3,MYC,PTGS2,TYROBP*	9.96E-08	1.109
	Quantity of Lymphoid tissue	*ARG1,CCR7,CXCR4,IL7,ITGB2,MAPK3,RAC1,TYROBP*	1.28E-06	1.06
	Cell Cycle Progression	*CSF2,HRAS,IL1B,IL7,MAP2K1,MAP2K6,MYC,PTGS2*	9.25E-05	0.599

*From IPA analysis

Table S6

Table S6- List of the genes that are significantly altered by HSD in small intestine lamina propria

Gene Code	Gene Name	Accession #	log2(ratio)	P value	Type
Cytokines, Chemokine and Receptors					
Il12a	*Interleukin 12A*	NM_008351.1	-4.56	0.0141	Cytokine
Il17a	*Interleukin 17A*	NM_010552.3	2.1	0.0381	Cytokine
Cxcr4	*C-X-C motif Chemokine Receptor 4*	NM_009911.3	-1.3	0.0464	Chemokine receptor
Ltb4r2	*Leukotriene B4 Receptor 2*	NM_020490.2	1.35	0.0139	Leukotrienes Receptor
Transcription Factors and Regulators					
Mafk	*V-Maf Avian Musculoaponeurotic Fibrosarcoma*	NM_010757.2	-0.42	0.0133	Transcription Factor
Mef2b	*Myocyte-specific Enhancer Factor 2B*	NM_001045484.1	-1.84	0.0201	Transcription Factor
Mef2c_Mm	*Myocyte-specific Enhancer Factor 2C*	NM_025282.2	-0.88	0.0316	Transcription Factor
Signal Transduction Elements					
Map2k4	*Mitogen-Activated Protein Kinase Kinase 4*	NM_009157.4	-0.15	0.0123	Signal Transduction Kinsases
Innate Immunity System					
Tlr1	*Toll-Like Receptor 1*	NM_030682.1	-0.7	0.024	Receptor
Tlr9	*Toll-Like Receptor 9*	NM_031178.2	-0.82	0.033	Receptor
Defa-rs1	*Alpha-Defensin-Related Sequence 1*	NM_007844.2	0.5	0.0371	Defensin

*From IPA analysis

Table S7

Table S7 - Most significant canonical pathways altered by HSD in small intestine lamina propria

Canonical Pathways	Genes involved	p-value	Ratio	z-score
**Small Intestine**				
Role of Pattern Recognition Receptors in Recognition of Bacteria and Viruses	*MAP2K4,IL12A,TLR1,TLR9,IL17A*	9.01E-09	3.79E-02	NaN
Toll-like Receptor Signaling	*MAP2K4,IL12A,TLR1,TLR9*	8.59E-08	5.56E-02	NaN
IL-17 Signaling	*MAP2K4,TLR9,IL17A*	1.76E-05	3.57E-02	NaN
Communication between Innate and Adaptive Immune Cells	*IL12A,TLR1,TLR9*	1.76E-05	3.57E-02	NaN
p38 MAPK Signaling	*MAP2K4,MEF2C,MEF2B*	3.95E-05	2.73E-02	NaN

*From IPA analysis

Table S8

Table S8 - List of genes that are statistically altered by DSS in Germ-Free mice

Gene expression was acquired using Nanostring technology, from a costumized gene panel

Gene Name	Gene Function	DSS*	DSS*	DSS*	Control*	Control*	Control*	log2ratio	p-value
GABAB	Neuro	616.07	735.68	632.93	189.68	172.53	168.23	1.9	0.0001
Tjp1	Barrier	2192.85	2423.08	2210.85	1410.66	1233.45	1305.33	0.79	0.0005
Cdh1	Barrier	18558.07	17560.81	16639.28	11999.26	12128.77	10918.29	0.59	0.0009
Mapk1	Immune	4223.89	4414.01	4148.45	3550.86	3416.6	3458.43	0.29	0.0016
Nfkb1	Immune	1645.58	1844.32	1874.55	1240.21	1098.16	1217.55	0.59	0.0019
NFAT	Immune	1502.39	1691.27	1592.82	1127.83	1105.94	1061.49	0.54	0.0023
Muc2	Barrier	57291.14	61718.74	55687.45	15352.86	10675.6	12250.46	2.21	0.0027
SEMAPHORIN	Neuro	198.83	234.09	189.73	86.14	83.96	112.14	1.15	0.0032
Tollip	Immune	1157.57	1243.7	1072.79	872.77	888.89	801.4	0.44	0.0059
GDNF	Neuro	63.05	119.63	97.31	31.85	16.8	21.11	2.01	0.0064
Tff3	Barrier	93229.47	109376.23	89628.82	61487.63	60132.8	47410.46	0.79	0.0076
P75	Neuro	156.04	154.35	198.64	96.24	80.07	107.27	0.84	0.0079
NOS	Neuro	266.32	375.56	266.57	163.16	154.04	108.89	1.09	0.0115
Il23r	Immune	52.35	43.74	23.81	6.59	2.2	8.11	2.95	0.019
Tlr4	Immune	867.07	964.61	940.27	446	537.53	621.77	0.8	0.0202
F2rl1	Immune	2594.45	2590.28	2353.39	2218.76	2074.39	1941.75	0.27	0.0204
Tlr5	Immune	73.74	83.61	101.76	52.05	59.63	40.62	0.77	0.0229
Hif1a	Immune	2278.43	2987.69	2397.93	4239	3347.49	3746.16	-0.57	0.023
CHAT	Neuro	46.59	74.61	31.61	17.96	6.09	5.67	2.49	0.0236
Rae1	Barrier	823.45	926.03	874.57	757.87	753.6	671.35	0.27	0.0248
Ocln	Barrier	3872.49	3750.37	3223.08	2606.39	2924.1	2203.47	0.49	0.0321
CALRET	Neuro	18949.8	16364.71	20667.03	11789.66	14783	14612.45	0.44	0.0376
CD11C	Immune	140.4	118.34	125.15	67.2	44.05	76.38	1.07	0.0377
Il22ra2	Immune	265.49	249.52	315.57	434.63	350.65	519.36	-0.64	0.0384
SOX10	Neuro	53.17	69.47	86.17	24.27	44.05	19.49	1.31	0.0441

* Normalized transcript counts

Table S9

Table S9 - Effect of HSD in Germ Free mice colonic gene expression

Gene expression was acquired using Nanostring technology, from a customized gene panel

Gene Name	Accession #	%CV**	Control Diet*	Control Diet*	Control Diet*	HSD*	HSD*	HSD*	log2ratio	p-value
NFAT	NM_010901.2	5.04	1204.52	1181.14	1133.67	1224.86	1299.2	1278.44	0.11	0.0366
Hif1a	NM_010431.2	22.36	4527.23	3575.11	4000.88	3097.03	2266.78	3356.97	-0.49	0.0874
Mapk1	NM_011949.3	4.56	3792.3	3648.91	3693.58	3373.96	3418.93	3649.66	-0.09	0.0998
Tff3	NM_011575.2	16	65668.45	64221.5	50634.1	49948.18	51113.29	43974.82	-0.31	0.1062
I-FABP	NM_007980.2	30.2	1342.06	1255.99	1019.95	2324.59	1504.1	1427.85	0.51	0.1339
Cldn2	NM_016675.3	7.69	1854.49	1969.08	1868.05	1578.47	1713.06	1863.82	-0.15	0.159
CALRET	NM_007591.3	11.63	12591.29	15788.17	15606.02	15721.58	17905.79	17265.02	0.22	0.1688
Muc2	NM_023566.2	19.3	16396.77	11401.49	13083.42	16955.2	14441.21	19648.53	0.33	0.1804
Tollip	NM_023764.3	10.42	932.12	949.33	855.89	868.96	838.99	698.17	-0.19	0.1805
Tlr5	NM_016928.2	18.4	55.59	63.68	43.38	49.6	40.53	42.17	-0.29	0.2171
P75	NM_033217.3	13.79	102.79	85.51	114.56	94.23	80.37	85.15	-0.21	0.2214
T-bet	NM_019507.1	90.07	5.69	1.04	2.58	1.14	1	1.02	-1.24	0.2229
GFAP	NM_001131020.1	28.26	82.56	61.6	45.98	45.03	54.35	40.12	-0.42	0.226
Cldn1	NM_016674.3	13.83	114.92	105.26	114.56	78.21	95.82	111.76	-0.24	0.2416
SOX10	NM_011437.1	29.76	25.92	47.05	20.81	45.03	38.9	44.21	0.54	0.2645
Cdh1	NM_009864.2	6.01	12815.14	12953.46	11660.67	11229.98	12488.97	11543.17	-0.09	0.2665
GATA-3	NM_008091.3	25.39	32.66	15.86	32.09	37.02	35.65	37.05	0.52	0.2697
GABAB	NM_019439.3	9.52	202.58	184.26	179.67	224.69	182.82	215.12	0.13	0.2893
Cnlp	NM_009921.2	67.37	9.74	1.31	3.45	11.84	3.94	12.49	1.24	0.2908
5HT	NM_010484.2	18.19	271.35	250.79	236.09	207.52	263.32	158.83	-0.3	0.293
VIP	NM_011702.2	24.32	729.84	897.36	507.79	584.02	567.41	504.75	-0.33	0.3007
NPY	NM_023456.2	99.59	1.35	5.47	1	1	1	1.02	-0.95	0.3356
CD11C	NM_021334.2	32.58	71.77	47.05	81.57	82.79	121.03	60.59	0.38	0.3703
NOS	NM_008712.2	17.69	174.26	164.51	116.3	205.24	163.31	154.74	0.21	0.3971
TGR5	NM_174985.1	106.61	5.69	1.04	1	1	1	1.02	-0.84	0.415
Myd88	NM_010851.2	16.97	437.22	504.43	610.22	477.59	682.06	604.02	0.18	0.4244
CALB	NM_009788.4	104.92	5.69	1.04	1	1.14	1	1	-0.79	0.4404
NR2B	NM_008171.3	5.43	1	1.04	1	1.14	1	1.02	0.06	0.4445
CCR6	NM_001190333.1	69.96	70.42	25.22	131.92	56.47	220.22	89.24	0.75	0.4559
HMGB1	NM_010439.3	7.16	1478.26	1496.11	1251.72	1325.56	1367.5	1303	-0.08	0.4572
Gpr44	NM_009962.2	58.53	3	1.04	1	1.14	1	1.02	-0.47	0.4606
CD11b	NM_001082960.1	35.93	25.92	32.5	69.42	40.45	60.86	52.4	0.38	0.4792
Rae1	NM_175112.5	6.4	809.4	804.84	717	707.61	725.97	800.51	-0.06	0.4798
F2rl1	NM_007974.4	7.77	2369.62	2215.44	2073.78	2242.19	2206.61	1880.2	-0.08	0.4898
Nfkb1	NM_008689.2	8.8	1324.53	1172.83	1300.33	1182.52	1365.87	1475.95	0.08	0.5125
Casp8	NM_009812.2	13.47	580.16	612.54	700.5	476.45	590.18	688.96	-0.12	0.5382
Il23r	NM_144548.1	43.88	7.04	2.35	8.66	8.41	10.45	4.3	0.47	0.547
Tlr4	NM_021297.2	26.33	476.32	574.07	664.04	452.42	899.15	697.14	0.21	0.5536
SUBP	XM_006505028.1	27.15	487.11	451.41	276.02	376.89	426.75	230.47	-0.24	0.5603
BDNF	NM_007540.4	88.97	1.65	1.04	1	4.97	1	1.02	0.52	0.571
Reg3g	NM_011260.1	54.64	40.76	8.59	41.64	22.14	63.3	29.89	0.51	0.6009
CGRP	NM_007587.2	12.48	1.35	1.04	1	1.14	1	1.02	-0.09	0.6038
Defcr1	NM_010031.2	12.48	1.35	1.04	1	1.14	1	1.02	-0.09	0.6038
GABAA	NM_010252.4	12.48	1.35	1.04	1	1.14	1	1.02	-0.09	0.6038
Il17a	NM_010552.3	12.48	1.35	1.04	1	1.14	1	1.02	-0.09	0.6038
PGP9.5	NM_011670.2	12.48	1.35	1.04	1	1.14	1	1.02	-0.09	0.6038
TAAR1	NM_053205.1	12.48	1.35	1.04	1	1.14	1	1.02	-0.09	0.6038
SEMAPHORIN	NM_011352.2	17.37	92	89.67	119.77	96.52	134.85	96.41	0.11	0.6128
S100BETA	NM_009115.3	49.2	11.09	8.59	9.53	17.56	11.26	2.25	-0.34	0.7414
NR2D	NM_008172.2	18.51	42.1	36.65	26.89	41.59	29.15	41.14	0.09	0.7462
Il22ra2	NM_178258.5	40.16	464.19	374.49	554.67	312.81	899.97	484.28	0.17	0.7512
CCR2	NM_009915.2	30.86	175.61	206.09	306.4	152.6	308.04	181.35	-0.13	0.7612
CXCR3	NM_009910.2	26.02	42.1	52.25	42.51	40.45	63.3	28.86	-0.11	0.7684
MMP9	NM_013599.2	33	92	72	36.44	73.64	51.91	47.28	-0.14	0.7757
Pparg	NM_011146.1	25.97	25.92	16.9	18.21	24.43	12.07	21.7	-0.11	0.7901
CHAT	NM_009891.2	73.29	19.18	6.51	6.05	23.28	1	14.53	-0.39	0.8176
trpv1	NM_001001445.1	40.53	11.09	7.55	6.05	11.84	3.13	10.44	-0.13	0.8585
Cxcr4	NM_009911.3	41.77	449.35	222.72	328.97	233.85	604.82	283.69	0.1	0.8623
Mylk3	NM_175441.5	123.04	12.44	1.04	1	1.14	6.38	1.02	-0.26	0.8677
TAAR4	NM_001008499.1	69.65	7.04	2.35	5.18	3.83	1.5	11.46	-0.13	0.9037
Lyz1	NM_013590.3	45.08	7008.48	7605.24	11796.09	5960.21	17006.51	7054.53	0.06	0.9136
Tlr2	NM_011905.2	35.18	100.09	84.47	162.3	81.65	178.76	102.55	0.04	0.9289
Tjp1	NM_009386.1	4.42	1506.58	1317.32	1394.09	1414.82	1381.32	1429.9	0	0.9409
C3	NM_009778.2	41.53	5196.09	2381.76	3592.89	3200.02	6301.34	2355.06	0.03	0.9555
Nod2	NM_145857.2	66.67	7.04	6.51	26.89	6.12	10.45	17.61	-0.04	0.9591
GDNF	NM_010275.2	38.74	34.01	17.94	22.55	41.59	17.76	19.65	0.03	0.9593
Ocln	NM_008756.2	17.75	2783.61	3122.92	2353.3	2423	3586.42	2406.23	0.01	0.9652
Cd86	NM_019388.3	38.99	62.33	33.54	63.35	35.87	89.32	43.19	0.02	0.9685

* Normalized transcripts counts

** Coefficient of variation percantage

Table S10

Table S10 - Fecal microbiota genera comparison between recipient mice groups

TAXONOMY: GENERA	Test-Statistic	p-Value	q (FDR)
Moryella	-3.738	0.013	0.229
Parabacteroides	2.577	0.050	0.419
Enterobacter	-2.255	0.074	0.419
Akkermansia	-1.768	0.137	0.525
Mucispirillum	1.630	0.164	0.525
Turicibacter	1.535	0.185	0.525
Lachnospira	-1.387	0.224	0.544
Oscillospira	-1.198	0.285	0.545
Ruminococcus	-1.187	0.289	0.545
Lachnospiraceae (Unclassified Genus)	-1.098	0.322	0.548
Lactobacillus	0.794	0.463	0.655
Eubacterium	-0.772	0.475	0.655
Clostridiales (Unclassified Genus)	0.725	0.501	0.655
Blautia	-0.173	0.869	1.000
Porphyromonadaceae (Unclassified Genus)	0.094	0.929	1.000
Enterococcaceae (Unclassified Genus)	-0.064	0.952	1.000
